# Foraging and recruitment hotspot dynamics for the largest Atlantic loggerhead turtle rookery

**DOI:** 10.1038/s41598-017-17206-3

**Published:** 2017-12-04

**Authors:** Simona A. Ceriani, John F. Weishampel, Llewellyn M. Ehrhart, Katherine L. Mansfield, Michael B. Wunder

**Affiliations:** 1 Florida Fish and Wildlife Conservation Commission, Fish and Wildlife Research Institute, Saint Petersburg, Florida 33701 USA; 2 Department of Biology, University of Central Florida, Orlando, Florida 32816 USA; 3 Department of Integrative Biology, University of Colorado Denver, Denver, Colorado, 80204 USA

## Abstract

Determining patterns of migratory connectivity for highly-mobile, wide-ranging species, such as sea turtles, is challenging. Here, we combined satellite telemetry and stable isotope analysis to estimate foraging locations for 749 individual loggerheads nesting along the east central Florida (USA) coast, the largest rookery for the Northwest Atlantic population. We aggregated individual results by year, identified seven foraging hotspots and tracked these summaries to describe the dynamics of inter-annual contributions of these geographic areas to this rookery over a nine-year period. Using reproductive information for a subset of turtles (n = 513), we estimated hatchling yields associated with each hotspots. We found considerable inter-annual variability in the relative contribution of foraging areas to the nesting adults. Also reproductive success differed among foraging hotspots; females using southern foraging areas laid nests that produced more offspring in all but one year of the study. These analyses identified two high priority areas for future research and conservation efforts: the continental shelf adjacent to east central Florida and the Great Bahama Bank, which support higher numbers of foraging females that provide higher rates of hatchling production. The implementation of the continuous-surface approach to determine geographic origins of unknown migrants is applicable to other migratory species.

## Introduction

Many marine species are difficult to study as they undertake ocean-wide developmental and breeding migrations during their life cycle^1^. Our understanding of the ecology of migrants that move between geographically distinct feeding and reproductive areas has been expanded by technological advances that are becoming progressively more affordable such as satellite telemetry, genetic markers and intrinsic markers [e.g., stable isotope analysis (SIA)^2,3^]. When these techniques are combined, they provide complementary information to unravel patterns associated with migratory connectivity^4,5^.

Sea turtles are long-living, late-maturing and highly migratory species of conservation concern that are primarily studied on nesting beaches where they are easily accessible. However, only a small portion of their complex life histories is spent in their breeding habitats. Reproductively active females undertake breeding migrations every 1 to 9+ years often from distant long-term residence areas (hereafter, foraging areas) to their natal nesting beach where they typically lay several clutches in a nesting season^6^.

Sea turtle status assessments and recovery plans^7,8^ rely heavily on long-term standardized annual nest counts. These are used as indirect indices of female abundance^9^. Even though nesting activities may have been monitored for decades, we cannot confidently identify the drivers of nest count trends because our understanding of demographic rates (i.e., breeding rates, clutch frequency) is imprecise and our characterization of the ecological context that influences demographic parameters (e.g., resource availability, temperature, oceanic current systems and oceanic productivity) is speculative^10^. Thus, abundance estimates, demographic parameters, genetic relationships among nesting populations, locations of commonly used foraging areas and related threats, effects of coastal and pelagic fisheries are critical data needs for sea turtle assessment and management^10^.

Satellite telemetry has contributed to understanding the spatial ecology of individual adult females and addressing some data needs by identifying foraging areas, migratory corridors and habitat use^5,11^. Though telemetry depicts detailed organism-level migratory paths, it is less suitable for understanding population-wide dynamics when individual movements are idiosyncratic, such as found with sea turtles, because typically only a few individuals are tracked in any given year due to high sensor costs, which can lead to biased or imprecise results. However, population-level questions can be addressed at a coarser spatial resolution using the comparatively cost-effective SIA, once the isotopic approach has been validated by satellite telemetry^12–14^. Stable isotopes are eco-geochemical markers that act as forensic recorders of migratory and foraging behaviors^3^. Tissue samples (e.g., egg-yolk, epidermis, red blood cells, scute, unhatched eggs) collected from nesting sea turtles represent an integration of diet and geographic location used prior to nesting^15,16^. The integration of these two approaches has augmented our knowledge of sea turtle migratory ecology^12,17^.

Post-nesting satellite-tracked females that migrate to different foraging areas can be identified by differences in their isotopic signatures. Adult females exhibit natal philopatry and fidelity to feeding areas throughout their adult life^16,18,19^. Thus, researchers have focused on sea turtle nesting aggregations by sampling nesting females and their nests and have used SIA to infer foraging areas of untracked individuals^13,14,20–22^. The potential of cost-effective SIA to elucidate nesting and in-water trends (e.g., changes in contribution of females from different foraging areas over time which may be related to differential survival probabilities at residence areas) has been denoted^22–24^. From isotopic signatures coupled with satellite geolocation data and in-water captures, researchers developed loggerhead-specific isoscapes (maps of stable isotope ratios) for the Northwest Atlantic (NWA) and found geographic discrimination in δ^13^C and δ^15^N suggesting that a spatially-explicit, continuous-surface approach may provide further insight into this species’ migratory patterns^23^. Isoscapes can be used to create geographic models for the probability of tissue origin^25^, which can in turn be used as a proxy of foraging locations. Population-level summaries of the probability models can help to identify foraging hotspots^24^.

We focused on loggerhead nesting at the Archie Carr National Wildlife Refuge (ACNWR), which accounts for ~14% of the nests laid by the NWA loggerhead rookery^26^, the largest subpopulation in the world^7^. Previous satellite tagging, flipper tag returns, and SIA have provided details on post-nesting female migration^14,27,28^ and which broad residence areas are used for foraging by this nesting aggregation^13,22,29^. However, a spatially-explicit approach has yet to be applied to infer origin of unknown (non-satellite tracked) high trophic level and migratory marine animals^30^. Here, we update previously developed isoscapes by including a larger number of known-origin (satellite-tracked) adult females and apply a continuous-surface likelihood approach to: 1) determine geographic histories for a large number of untagged nesting loggerheads; 2) identify geographic comparatively persistent foraging hotspots; 3) examine the dynamics of inter-annual contribution of foraging areas to this rookery over a nine-year period (2007–2015), and 4) evaluate geographic patterns associated with female foraging as weighted by the proportion of hatchlings that emerge from their respective nests.

## Results

We created loggerhead-specific δ^13^C and δ^15^N isoscapes (Supplementary Fig. S1) and used a bivariate normal model to assign a probability of origin to each raster cell for every individual female turtle in the dataset. The median of the normalized posterior probabilities for raster cells associated with known latitude-longitude coordinates of known-origin turtles (*calibration dataset*) was 0.92; the value of the first quartile of modeled probability values for known locations was 0.78 (Supplementary Fig. S2). We modeled an evidence-based index of foraging area importance for the entire dataset of unknown females (n = 749) as well as for the subset of females for which we had nest fate information (n = 513 females) (Supplementary Table S3). The resulting maps of the relative importance of foraging geographies (*foraging indices*) were similar for the two data sets; here, we present only the results for the latter subset (n = 513 females) that includes the reproductive data.

**Figure 1:**
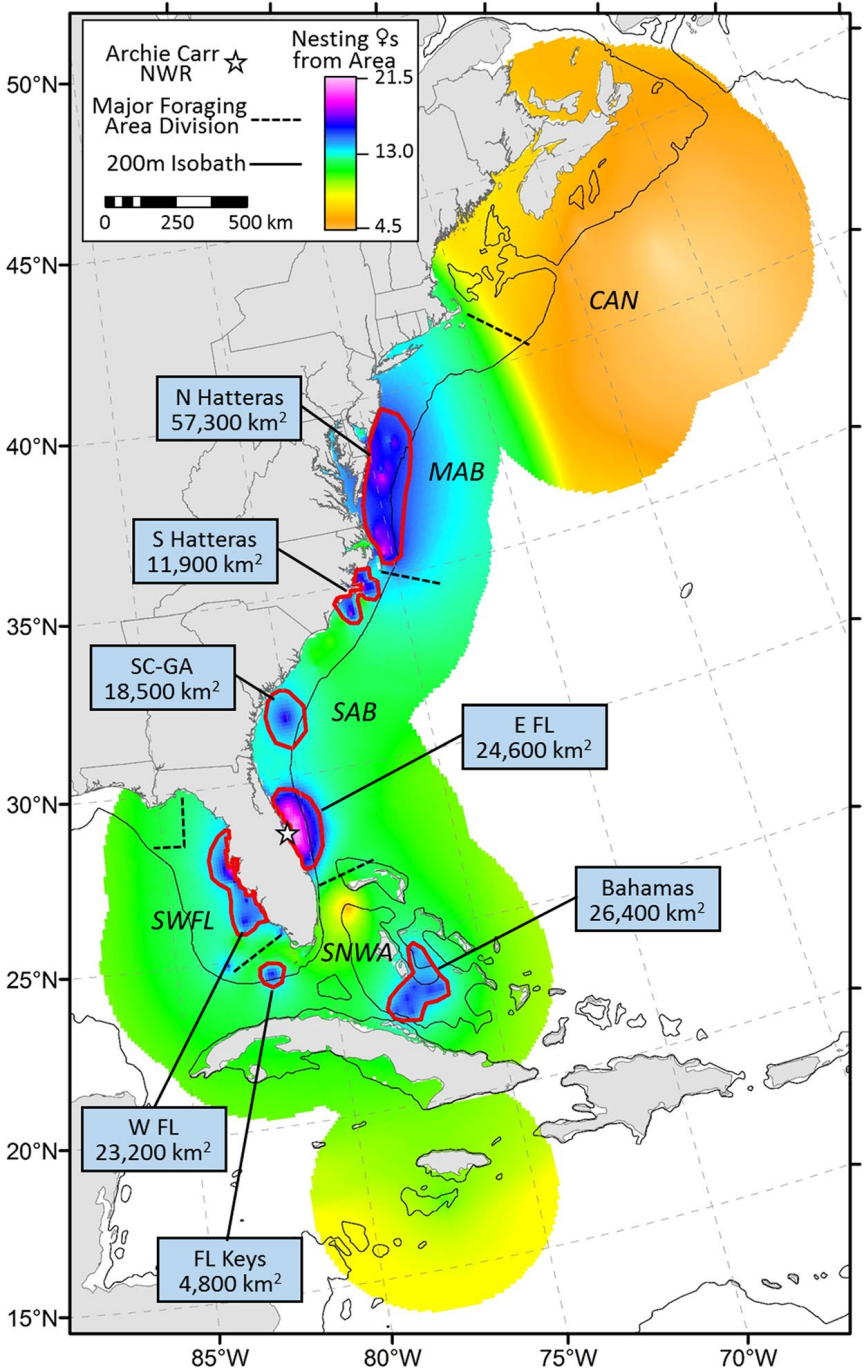
Population-level summary representing relative contribution of foraging regions to ACNWR (Archie Carr National Wildlife Refuge) based on estimated nesting female (♀) numbers averaged over nine years (2007–15). Broad geographic areas used by NWA loggerheads: CAN (waters off Nova Scotia, Canada), MAB (Mid-Atlantic Bight), SAB (South-Atlantic Bight), SNWA (Subtropical Northwest Atlantic), SWFL (Southwest Florida continental shelf). The seven hotspots identified by this study are outlined in red: N Hatteras (North Hatteras), S Hatteras (South Hatteras), SC-GA (South Carolina/Georgia border), E FL (east central Florida), Bahamas (continental shelf south of Andros), FL Keys (Florida Keys), and W FL (continental shelf on west coast of Florida). Map was created using ArcGIS v. 10.2 (http://www.esri.com/software/arcgis).

**Figure 2:**
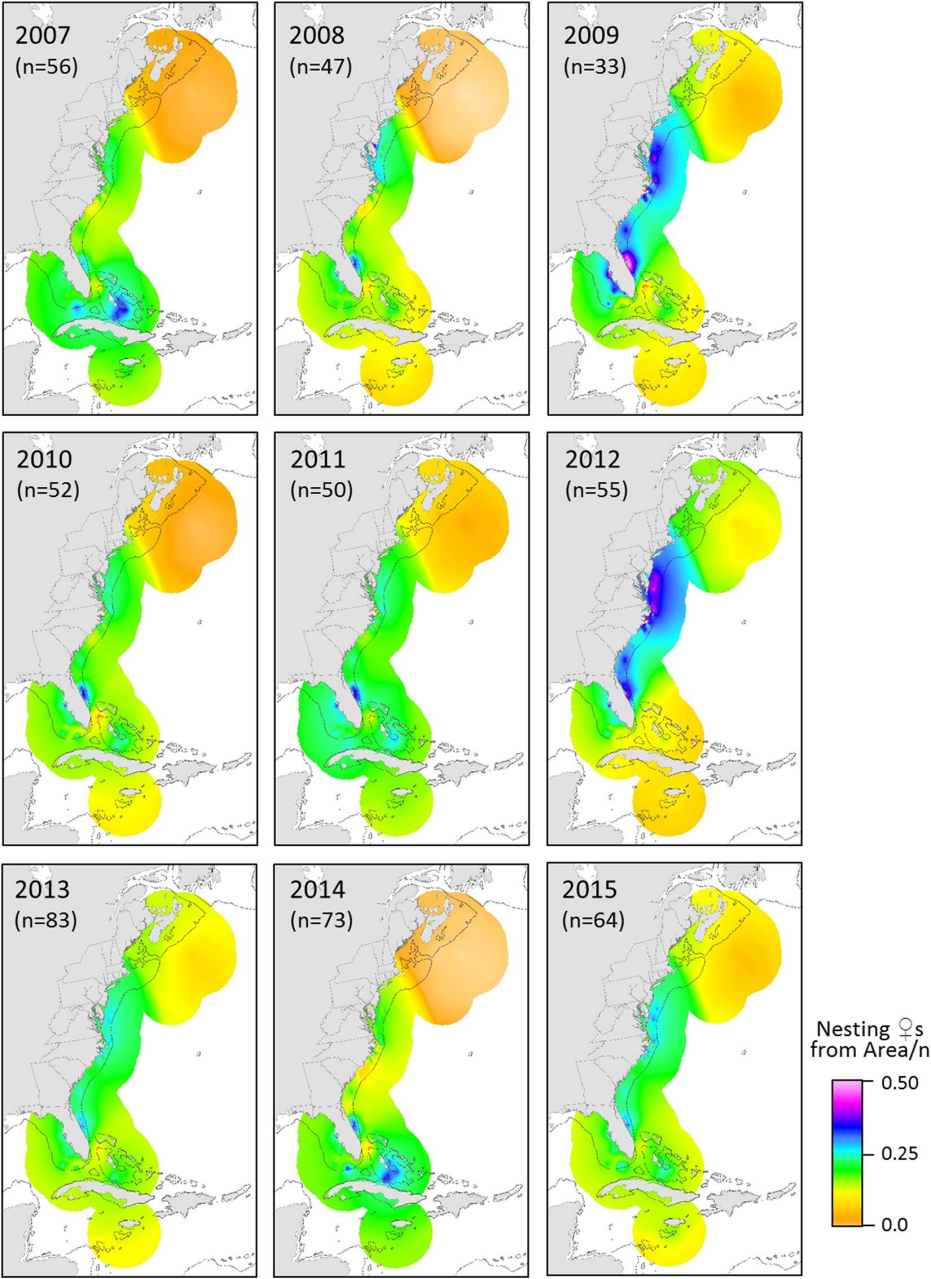
Annual population-level summaries representing relative importance of foraging areas based on estimated nesting loggerhead (♀) numbers present in the region divided by sample size (n). The solid gray line represents the 200 m isobaths. Maps were created using ArcGIS v. 10.2 (http://www.esri.com/software/arcgis).

This approach allowed the assignment of all individuals in the unknown dataset and the compilation of overall and annual population-level summaries (Figs 1 & 2). Seven foraging hotspots were consistently identified by the modeled foraging index for loggerheads nesting at the ACNWR (Fig. 1). These consist of the waters: (i) centered around the Delmarva Peninsula (N Hatteras); (ii) along the continental shelf next to North Carolina (S Hatteras); (iii) adjacent to the South Carolina/Georgia border (SC-GA); (iv) adjacent to east central Florida (E FL); (v) on the continental shelf south of Andros in the Great Bahama Bank (Bahamas); (vi) near the distal portion of the Florida Keys (FL Keys), and (vii) along the continental shelf from the west coast of Florida centered around Tampa Bay and Charlotte Harbor (W FL).

**Figure 3:**
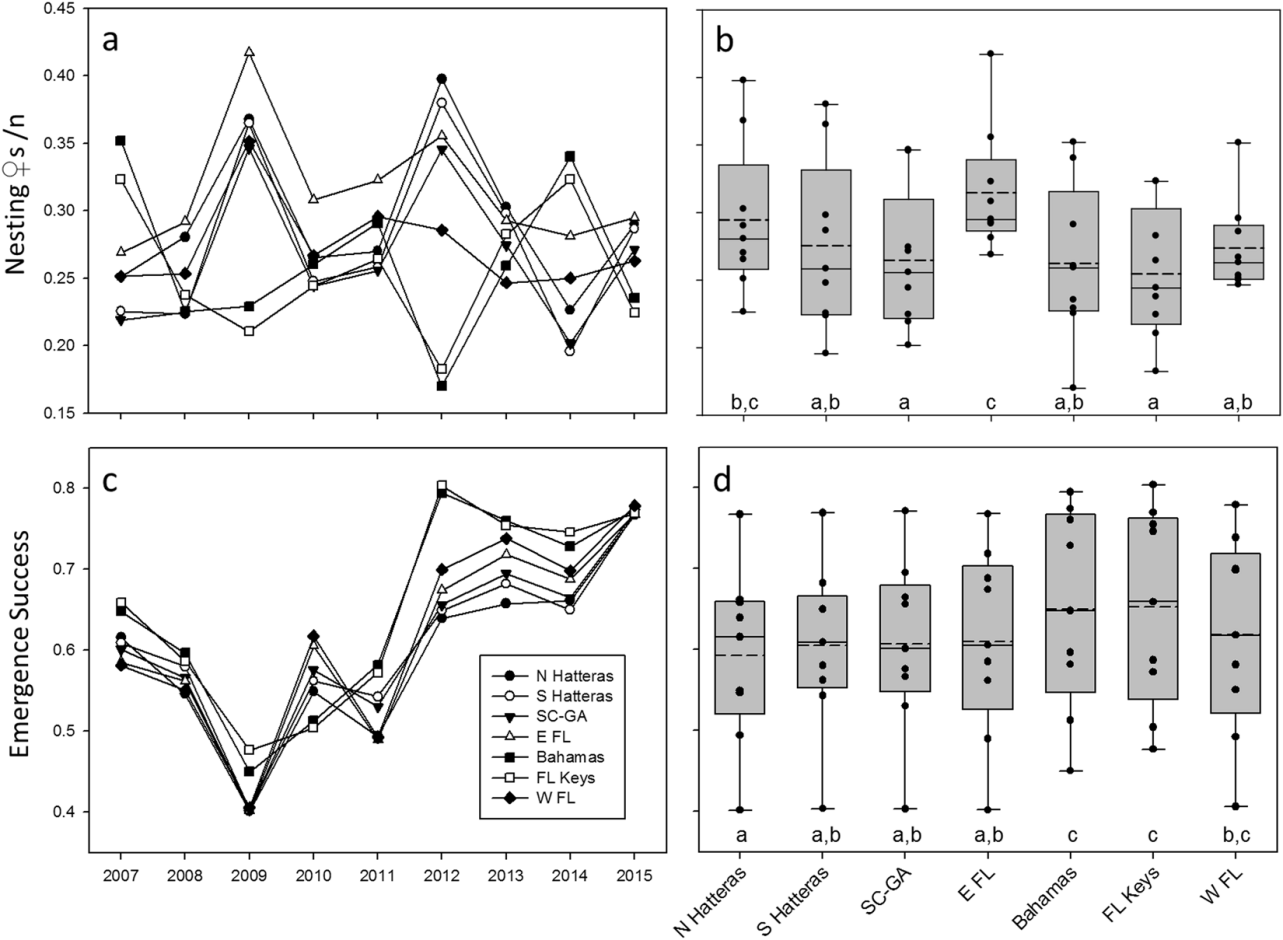
Annual (**a**,**c**) and average (**b**,**d**) importance of the seven foraging hotspots associated with nesting female (♀) numbers (**a**,**b**) and emergence success (**c**,**d**) at ACNWR. Boxes reflects quartiles, whiskers extend to the tenth and 90^th^ percentile. Dashed and solid lines indicate mean and median, respectively. Black dots indicate the nine annual nesting (**b**) and emergence success (**d**) values for each foraging hotspot (summarized from panels a and c, respectively). Hotspots are N Hatteras (North Hatteras), S Hatteras (South Hatteras), SC-GA (South Carolina/Georgia border), E FL (east central Florida), Bahamas (continental shelf south of Andros), FL Keys (Florida Keys), and W FL (continental shelf on west coast of Florida). Lower-case letters below the boxplot for each foraging area resulted from post-hoc pairwise hypothesis tests for no difference in ranked sum of mean number of females or emergence rate between each pair of foraging areas. Any pair that does not share the same letter indicates that the hypothesis test for no difference in ranks resulted in a p-value < 0.05.

We found conspicuous inter-annual variability in the relative values of the foraging area indices for the ACNWR nesting aggregation (Fig. 2). Some foraging hotspots were synchronous (i.e. appeared to contribute similar proportions of nesters in a given year) while others exhibited the opposite pattern (Fig. 3a). The southern hotspots (Bahamas and Florida Keys) contributed similarly in each given year (high contributions in 2007 and 2014; low in 2009 and 2012). We found similar synchronous patterns for northern (North and South Hatteras) areas. Pulses of contributions from northern foraging areas (in 2009 and 2012) corresponded to a drop in apparent use of southern hotspots and vice versa. When the relative contribution values of southern foraging areas were highest (2007 and 2014), northern hotspot index values were at their lowest. The comparative apparent use across the seven foraging hotspots was similar for the five remaining years (2008, 2010, 2011, 2013 and 2015). Lastly, the relative contributions of the foraging hotspot adjacent to the nesting beach (east central Florida) appeared consistently high and fairly stable over time (mean = 0.31, range: 0.27–42), indicating regular use by a large portion of females nesting at this critically important site. The overall population-level hypothesis test for no difference in the proportional distribution of use by nesting females across the seven hotspots, pooled over all nine years, suggested that the proportional use was not uniform (Friedman *Χ* ^2^_(6)_ = 18.668, P = 0.005; Fig. 1 & Fig. 3b). Post-hoc pair-wise null hypothesis tests for the same uniformity, and examination of the data shown in Fig. 3a,b suggested that the east central Florida foraging area was used by more nesting females than all other foraging areas except for North Hatteras.

**Figure 4:**
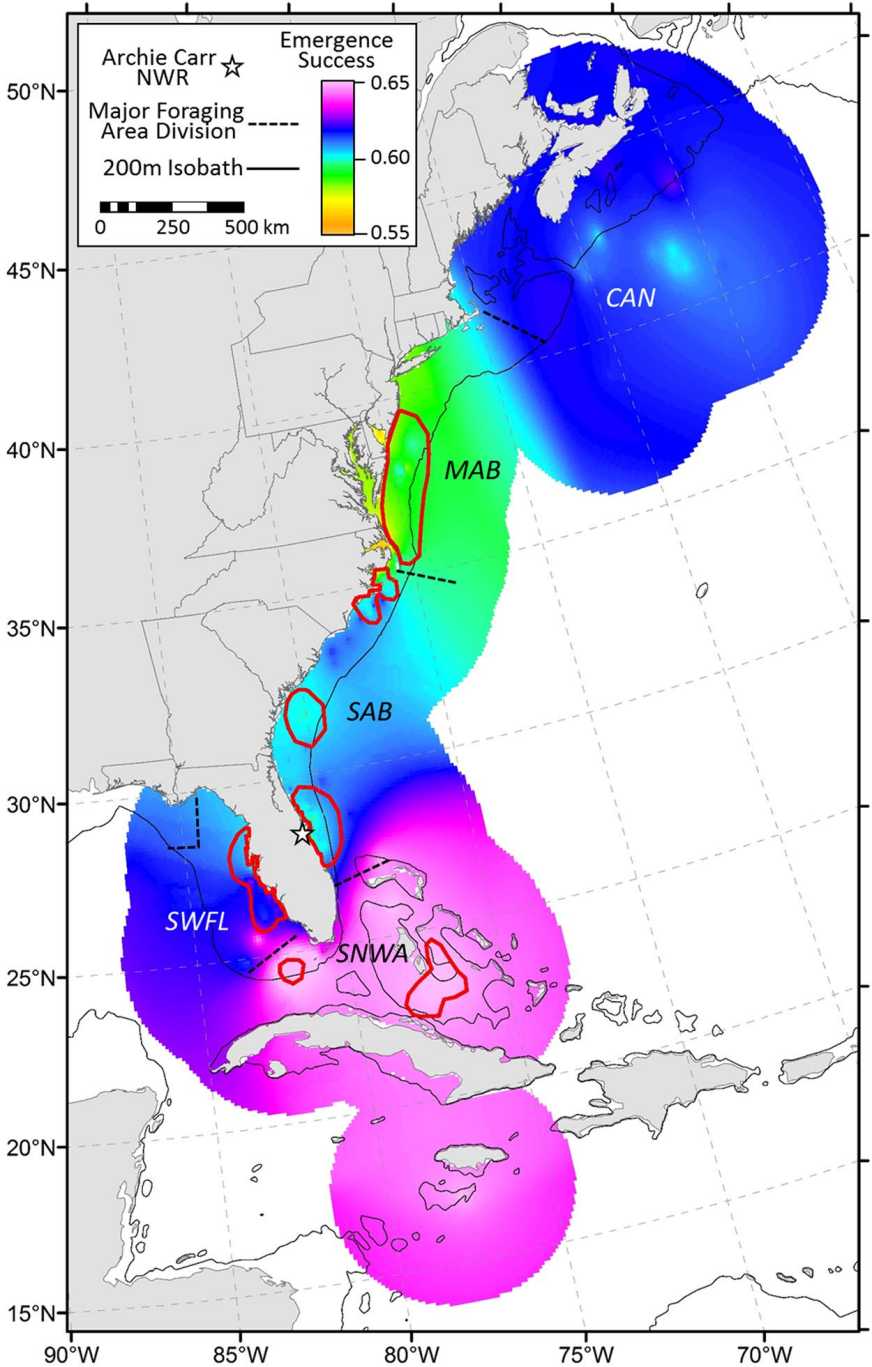
Population-level summary representing relative contribution of foraging regions to reproductive output at ACNWR based on estimated emergence success averaged over nine years (2007–15). The seven designated hotspots are outlined in red. Map was created using ArcGIS v. 10.2 (http://www.esri.com/software/arcgis).

**Figure 5:**
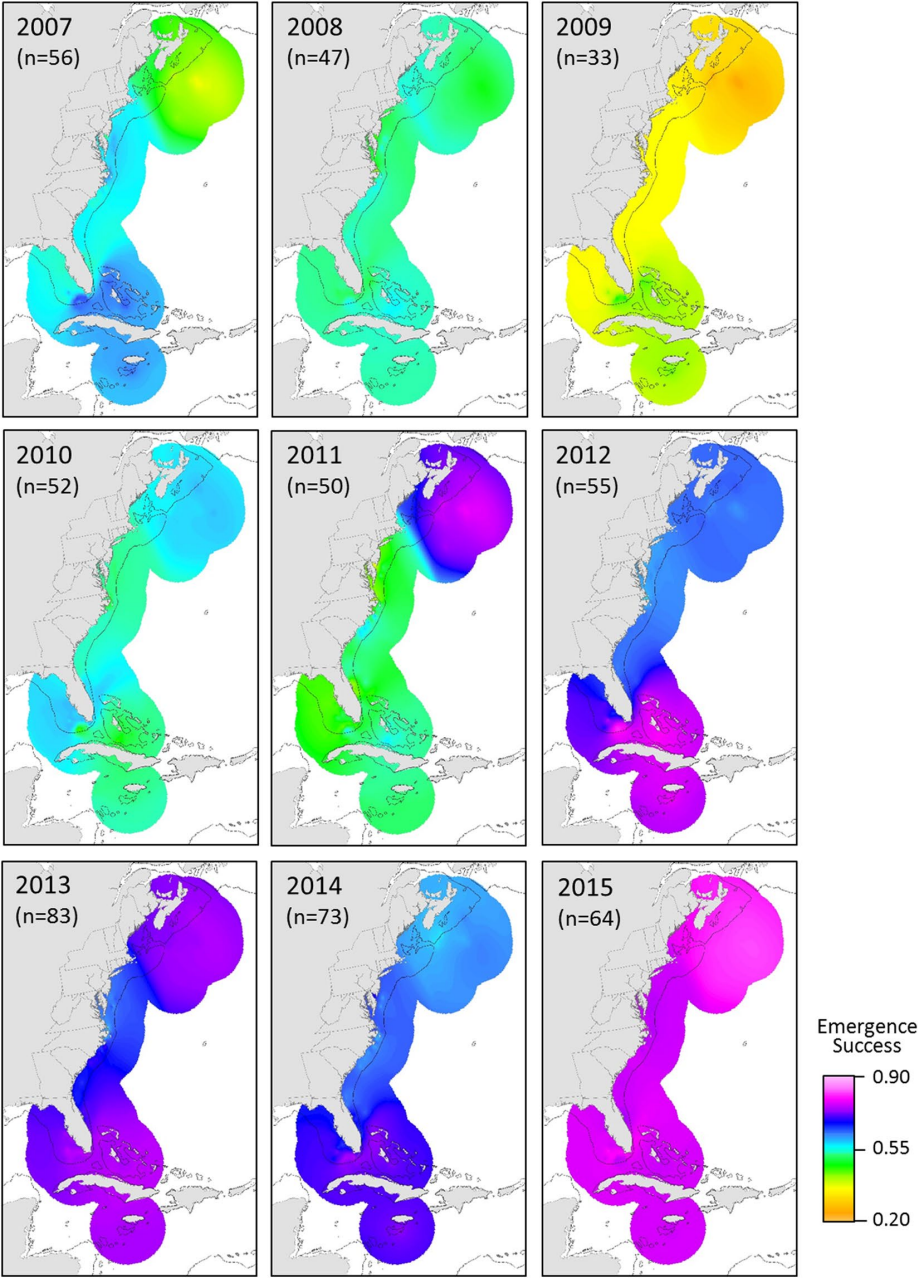
Annual population-level summaries representing relative contribution of foraging regions to reproductive output at ACNWR based on estimated emergence success. Maps were created using ArcGIS v. 10.2 (http://www.esri.com/software/arcgis).

Examining the overall patterns, we found agreement among average relative importance of foraging areas to the nesting aggregation based on nesting females (Fig. 1), egg and emergent numbers (Supplementary Fig. S4). Foraging areas used by large numbers of breeding females provided nutrients and energy for high egg production and in turn yielded high numbers of viable offspring. Examination of the data and results from the hypothesis test for no difference in the proportional distribution of rate of emergence associated with female foraging areas both suggested that the distribution of emergent production was likewise not uniform (Fig. 3d, Fig. 4; *Χ* ^2^_(6)_ = 22.762, P = 0.001); however, the patterns of deviation from uniformity differed from those for the mean number of nesting females. Nests laid by females that used the northernmost foraging hotspot (North Hatteras) had lower emergence success than females foraging at lower-latitudes (Bahamas, Florida Keys and West Florida; Fig. 3d). Overall, females foraging in the Bahamas and Florida Keys laid nests with higher mean annual emergence success rates (Fig. 4). There was notable geographic patterning in relative importance of foraging areas to emergence success within and among years. Overall emergence success was relatively high in some years (2012–2015), intermediate in others (2007–2008, 2010–2011) and low in 2009, regardless of the foraging area females used prior to each nesting season (Fig. 3c & Fig. 5). Within-year differences in emergence success among foraging hotspots were comparatively low. Nevertheless, our results indicated that females foraging in the southern hotspots (the Bahamas and FL Keys) showed consistently higher emergence success than the other hotspots for all but one year (2010) (Fig. 3c).

## Discussion

The results of this study are particularly relevant from a conservation standpoint because it focuses on the major nesting aggregation in the Atlantic and determined likely geographic origin for a large number of truly unknown loggerheads (n = 749) over nine years (2007–2015). The continuous-surface approach allowed the inclusion of all encountered individuals, improving previous assignment models to origin in sea turtles^13,14,20–22^. The probabilistic assignment to origin with isoscapes has been used in terrestrial systems to track dispersal and aggregate individual results to identify geographic hotspots at the population level^31–34^. Though this method has been validated in few marine systems^24,30^, it has not been previously implemented to infer origin of unknown individuals.

The geographic locations of the seven foraging hotspots identified by the model agreed with available telemetry data^14,27,28^ and flipper tag returns^29,35^, while providing novel information on their relative contributions (within and among years) to the ACNWR nesting aggregation. Our findings based on a more representative sample of this rookery (i.e., dozens of individual females sampled annually across the entire nesting season) showed that the continental shelf off east central Florida consistently contributed the most nesting females to the ACNWR. Females residing year-round in east central Florida have access to moderately high productive waters^36^ and incur low energetic costs associated with migration allowing them to invest more resources in reproduction, thereby increasing overall reproductive success. However, the importance of east central Florida as a year-round foraging hotspot has been underestimated by satellite telemetry studies involving post-nesting loggerheads (11%, n = 6 of 56 females)^14,27,28,37^. Evidence supporting a greater importance of the middle and outer continental shelf off east central Florida as a year-round foraging hotspot for adult loggerheads is based on a satellite telemetry study of adult male loggerheads (33%, n = 8 of 24 individuals)^38^ and to some extent on long-term stranding records (FWC unpublished data) and aerial surveys^39^. The low percentage of satellite-tracked nesting females reported to reside in this area may be an artefact of small telemetry sample sizes in a given year (4 females tracked/year on average) or time of deployment. Telemetry studies conducted at the ACNWR aimed to identify female post-nesting migration destinations; thus, satellite tag deployment was temporally biased toward the end of the nesting season (end of July and August). Turtles migrating from distinct foraging areas may differ in their nesting phenologies (i.e., time of arrival and departure from the nesting area); hence, the combination of a small sample size and temporally biased sampling may be responsible for underestimating the importance of east central Florida for this rookery. Previous work relying on SIA to infer foraging areas used by loggerheads nesting at the ACNWR produced conflicting results on the importance of east central Florida as a foraging hotspot despite sharing common methodology (nominal approach, e.g., Discriminant Function Analysis) with the only difference being the number of isotopically distinct foraging areas of origin used in the assignment model (3 vs. 4)^13,14,22^. Here, the continuous-surface approach overcame limitations of previous sea turtle isotopic research: boundary definition of possible source of origin, low spatial resolution, and inability to assign all individuals with adequate accuracy.

We found considerable inter-annual variability in relative importance of foraging areas to the nesting aggregation suggesting plasticity in migratory connectivity linkages between foraging areas and the nesting site. Some years showed weak connectivity (i.e., all foraging areas contributed similarly as in 2013), while others were characterized by relatively strong connectivity (i.e., pulses of females coming either from the northern or the southern hotspots as in 2012 and 2014, respectively). The opposite pulse observed in some of the years suggests that females experience different environmental conditions at the two latitudinal extremes. Moreover, hotspots geographically close to each other (the Florida Keys and the Bahamas; North and South Hatteras) tended to act in synchrony. These similarities are likely the consequence of geographic proximity and shared marine ecoregions^36^. Females undertake breeding migration every 1 to 9+ years^6^ but most individuals return to their natal beach every two or three years^40,41^. The reverse pattern in some years (e.g. 2012 vs. 2014) indicated that many females were on a similar breeding schedule on those years. However, over the nine-year survey there were no clear patterns of periodicity of contributions from different foraging areas to the rookery suggesting variability in remigration interval (the number of years between successive nesting seasons for a particular individual) among females. The complexity in the migratory linkages we observed could be related to inter-annual environmental variability at the turtle’s foraging areas which is known to affect remigration intervals^42–44^. Models linking climatic variables and remigration intervals have not been developed for loggerheads nesting in the NWA, even though the NWA hosts the largest nesting population in the world for this species^7^. Understanding mechanistic links between climate and reproductive ecology will aid interpretation of trends in annual nest counts, which are the metric used to determine population status and set recovery goals (e.g., IUCN Red List Assessments, NMFS & USFWS recovery plans).

The relative importance of individual hotspots varied from year to year over the nine-year study. This result differs from previous isotopic and telemetry studies that found strong connectivity for NWA loggerhead females but were based on fewer years or small sample sizes of satellite tags deployed in any given year^13,20,22,28,45^. Our results suggest that overall the aggregation of females nesting at the ACNWR has relatively weak migratory connectivity, which could confer some level of resilience in light of climate change and other stochastic or anthropogenic threats. However, nine years represent only a short period of time for this long-living species—a fraction of a loggerhead generation. We emphasize the importance of conducting long-term studies to understand sea turtle migratory connectivity and reproductive ecology because nest counts fluctuate widely and females have variable remigration intervals; thus, decades are required to capture long-term trends.

NW Atlantic loggerheads undertake ocean-wide developmental migrations during their life cycle that can complicate conservation efforts^46^. The identification of adult foraging hotspots and understanding foraging area dynamics are critical to the development of effective conservation measures. The continuous-surface approach model identified the waters in the Great Bahama Bank (south of Andros and North of Cuba) as the only important foraging hotspot for the ACNWR rookery outside the USA Economic Exclusive Zone (EEZ), potentially simplifying strategies for the conservation of this critically important nesting aggregation. Telemetry as well as previous isotopic work revealed that the Great Bahama Bank is also an important foraging residence area for adult females from other genetically distinct sub-units within the NWA loggerhead subpopulation^20,22,24,45,47,48^. Yet, to our knowledge no research has been directly conducted on this important loggerhead foraging aggregation. Commercial fishing takes place on the continental shelves of the Bahamas targeting mostly spiny lobsters, snappers and conch^49^. Loggerheads mostly forage on these benthic invertebrates; hence, the Bahamas fishery could affect this loggerhead population both directly (e.g., causing mortality due to entanglement in fishing gears) and indirectly (e.g., by decreasing food availability). Harvesting sea turtles has been illegal in the Bahamas since 2009, but law enforcement is limited due to the vast marine area to patrol^48^. Our data identify the Great Bahama Bank south of Andros as a hotspot for adult loggerheads; we encourage the development of in-water capture programs to characterize this foraging aggregation.

The seven foraging hotspots identified in this study supported large numbers of breeding females thereby providing nutrients and energy for a large number of eggs and hatchlings. Assuming that our sampling of single nesting events were representative for a turtle that is expected to produce multiple clutches over a nesting season, the population-level summaries indicated that reproductive success differed among hotspots. Southern foraging areas yielded higher emergence success. In contrast, nests laid by northern foraging females yielded proportionately fewer emergents resulting in more net inflow of energy and allochthonous nutrients to the oligotrophic beach ecosystem^50,51^. Prior telemetry showed that most NWA loggerheads exhibit three main foraging strategies (seasonal-large scale, seasonal small-scale and a year-round). Females using the northernmost foraging hotspot (North Hatteras) undertake seasonal shelf-constrained movements between summer and winter areas^14,45^, loggerheads foraging in the South Atlantic Bight, where three of the hotspots we identified are located (South Hatteras, South Carolina-Georgia border and east central Florida), move short distances along the western edge of the Gulf Stream^45^, while females using the southernmost areas (Bahamas and Florida Keys) reside there year-round. Hence, northern foraging females incur higher energetic cost of migration compared to the other groups and the lower reproductive success of their nests perhaps may be related to differential allocation of energy within the reproductive component (i.e., migration vs. egg development cost). Although, egg quality was shown not to vary among females using different foraging areas and nesting in Japan^52^, the relationships between egg quality, foraging area location and energetic cost of migration have not been investigated for NWA loggerheads. On the other hand, previous isotopic work found that loggerheads foraging near large coastal estuaries at high latitudes have δ^15^N values higher than expected that may be associated with agricultural runoff and anthropogenic waste^23^. We hypothesize differences in egg loss and, thus, nutrient transfer among foraging areas may be a result of differential exposure to contaminants such as persistent organic pollutants (POPs), often used in agriculture, that could lead to reduced emergence success. While egg quality does not seem to vary among females using different foraging areas^52^, maternal transfer of POPs has been documented in sea turtles^53^ and POPs have been shown to affect embryonic mortality^54,55^. Thus, if POP concentrations differ among hotspots and females foraging in northern areas are exposed to higher concentrations, their eggs will likely have a lower survival. To our knowledge, no study has measured contaminant concentration in satellite tagged post-nesting females, although Alava *et al*.^56^ provided indirect support to our hypothesis, and differences in POP exposure among loggerheads using geographically distinct foraging areas were determined in adult male loggerheads in the NWA^57^.

We found considerable inter-annual variability in relative importance of foraging areas to emergence success. Regardless of the foraging area, there were relatively high years of emergence success (2012–2015) and 2009 was unusually low. Several studies have demonstrated that hatchling productivity (egg development and hatchling emergence) is affected by prevailing local climatic conditions (e.g. precipitation regimes and hurricane events), variations in nest microclimate and predation^58–61^. We attribute the 2009 overall low emergence success to the unusually high raccoon (*Procyon lotor*) predation rate documented in the ACNWR in that year (n = 21 out of 169, 12% of the nests included in the nest productivity assessment, FWC unpublished data) compared to the other years of the study (average 5%, SD ± 4%). In addition, previous isotopic studies found no differences in reproductive success among females utilizing different foraging areas^13,20^, and no differences in egg size and nutritional components were found between oceanic and neritic foraging loggerheads in the Pacific Ocean^52^. Although within-year differences in emergence success associated with different hotspots were generally low, emergence success was consistently slightly higher for nests laid by females foraging in southern areas for all the years of the study but 2010. We hypothesize that loggerheads that feed in the Bahamas and Florida Keys may have greater prey availability of higher nutritionally quality and/or be exposed to lower concentrations of contaminants which could result in higher emergence success.

Using isoscapes, we were able to derive population-level information to gain a better understanding of migratory connectivity for a critically important loggerhead nesting aggregation and inform managers on where to focus efforts to maximize conservation output. Specifically, our study identified two areas that should be prioritized for future research and conservation efforts: (i) the middle and outer continental shelf in east central Florida, a previously overlooked area and (ii) the continental shelf south of Andros in the Great Bahama Bank. The latter is the only major hotspot we identified outside the US EEZ and also appears to yield higher numbers of emerging hatchlings. The importance of these two hotspots as year-round foraging areas for loggerheads should be further investigated using tools such as aerial surveys, visual transects and in-water captures, increased telemetry effort, and interviews with commercial and recreational fishermen outside of the breeding season. A representative isotopic sample of the annual nesting population could be used in the future to (1) interpret trends in abundance at nesting beaches and demographic parameters affecting those trends and (2) monitor foraging aggregations trends from nesting beaches. We emphasize the importance of a long-term and intensive sampling for stable isotope analysis on nesting beaches combined with periodic telemetry studies to evaluate temporal isotopic consistency and prevent erroneous conclusions. Ultimately, the ACNWR is a good indicator rookery for monitoring the relative health and trends of the NWA loggerhead subpopulation because this 21+ km stretch of beach hosts 14.7% of the mean annual Florida loggerhead nest totals, yet its length represents only 1.6% of the surveyed beaches (FWC/FWRI Statewide Nesting Beach Survey Program database as of 20 February 2016). Hence, a better understanding of the movements and origins of loggerheads nesting at ACNWR may allow managers to identify potential areas of interaction with anthropogenic activities such as those associated with fishery operations and oil exploration. This study represents the first implementation of probabilistic assignment of origin with unknown individuals of a highly migratory marine species. Even though we used loggerhead-specific isoscapes, similar methodology could be applied to other migratory species.

## Methods

This study was performed in accordance with the guidelines of the University of Central Florida Institutional Animal Care and Use Committee (IACUC) and the Florida Fish and Wildlife Conservation Commission. The animal use protocol for this research was reviewed and approved by the University of Central Florida Institutional Animal Care and Use Committee (IACUC protocols #09–22 W, #12–22 W, #13–22 W, #15–13 W). Procedures were approved under the Florida Fish and Wildlife Conservation Commission (Marine Turtle Permit #25, #186 and #200).

### Study site

This study was conducted on the Brevard County portion (21+ km) of the ACNWR (27.917° N, 80.483°), a critically important loggerhead nesting beach in east central Florida. Here, all nesting activity has been continually monitored since 1982, and a subsample of females is encountered and tagged using both Inconel flipper tags and passive integrated transponders during annual nighttime surveys^41^.

### Female data, sample collection, and stable isotope preparation

A total of 749 loggerhead females were sampled for SIA (Supplementary Table S3). To avoid pseudoreplication, each turtle was tagged with Inconel flipper tags and, starting in 2009, with passive integrated transponders; thus, each female is represented only once in the dataset. Nighttime tagging effort was constant and sampling for this study spanned the nesting seasons (May–August) and the 21 km of ACNWR beach from 2007 to 2015. A subset (n = 513) of nests laid at the time of sampling were left *in situ* and marked for post-hatching nest content evaluation^62^ (Table 1). Tissues (skin and blood from nesting females, and contents of unhatched eggs from females tagged at night) were collected following methods described in a previous study^13^. These tissues have known isotopic relationships^13,15^ and are assumed to represent the isotopic signature of foraging areas used during the non-breeding season^12,14^.

**Table 1:** Number of loggerhead females sampled and nests from a fraction of the females whose clutch size and emergence success was recorded at the ACNWR over the nine-year period.

**Year**	**Females**	**Nests**
2007	63	56
2008	71	47
2009	58	33
2010	70	52
2011	73	50
2012	103	55
2013	98	83
2014	73	73
2015	140	64
**Total**	**749**	**513**

Samples were prepared for isotopic analysis following standard procedures^13,15^. Prepared samples were sent for mass spectrometry analysis to the University of South Florida, College of Marine Science. Replicate measurements of internal lab reference materials (1577b Bovine liver) were used to estimate analytical precision and yield a precision (reflecting ± 1 SD) of ±0.16‰ for δ^13^C and 0.17‰ for δ^15^N. All tissue isotopic values were converted to a common currency (epidermis) using established regression equations^13,15^. Epidermis samples were available for 543 females. Epidermis isotopic values were derived from unhatched egg and red blood cell values for 156 and 50 females, respectively.

Clutch sizes were determined either within 12 hours of deposition or at time of post-hatching excavation. The number of hatchlings that emerged from an individual nest (hereafter, “emergents”) was determined at the time of post-hatching excavation and was calculated as: # of hatched eggs − (dead hatchlings found in nest + live hatchlings found in nest). We followed established protocols used to evaluate nests post-hatching^62^. We included all 513 nests in the assessment of hatchling production, including those that were disturbed by storm-induced erosion or predators if the initial clutch size was recorded at the time of deposition. By doing so, we provide an unbiased representation of the female investment in the specific nesting event (expressed as clutch size) and reproductive output (indicated by the number of hatchlings that emerged). Loggerheads lay several clutches^63^ during a nesting season and the high number of nests at the ACNWR (12,350 nests/year, 5-year average from 2011 to 2015) prevented us from identifying and following the fate of all the nests laid by individual females. Thus, we did not investigate the investment of individual females during the entire nesting season but used the nest deposited the night of sampling as a proxy for that year female’s reproductive output. We then modeled expected number of eggs and emergents to explore annual variation in egg and hatchling production associated with geographic foraging hotspots.

### Computation of raster cell probabilities of use by individual loggerheads

We updated and re-analyzed the dataset comprised of 205 known-origin loggerheads^23^ that were sampled for SIA. An additional 22 females^13^ were equipped with satellite tags after nesting on Florida beaches, sampled for SIA, and augmented the known foraging areas used by loggerheads nesting at the ACNWR. This updated dataset (n = 227) was used for calibration. The isotopic values of these known-location loggerheads were used to develop carbon and nitrogen loggerhead-specific isoscapes (Supplementary Fig. S1) based on the empirical Bayesian kriging interpolation EBK^64^; routine available in ArcGIS 10.2 (Esri Redlands, CA) following previously developed procedures^23^. We used the resulting δ^13^C and δ^15^N isoscapes and assumed a bivariate normal distribution for the error term in the isotope model for assigning probability of foraging area origin. We parameterized this distribution independently for each raster cell in the study area. We used the predicted δ^13^C and δ^15^N from the EBK as the vector of means, and we estimated the variance-covariance matrix for δ^13^C and δ^15^N by combining information on tissue δ^13^C and δ^15^N from loggerheads that could be linked to known foraging areas from satellite data and in-water captures (sampling-based variances) and the rasters for the kriging errors (model-based variances). Specifically, we estimated the variance-covariance matrix for δ^13^C and δ^15^N from tissue samples at each of the six broad foraging areas identified from satellite data and in-water captures^13,23^: Canada (CAN), Mid-Atlantic Bight (MAB), South Atlantic Bight (SAB), Subtropical Northwest Atlantic (SNWA), Florida Keys (FL Keys) and Southwest Florida (SWFL). From these foraging-area specific matrices, we computed the average among-turtle correlations and variances, which were in turn combined with the kriging model variances and covariances for each raster cell. ArcGIS produced a raster of standard errors from the kriging model described above. We squared these values to approximate variances and added them to the mean sample-based variances for δ^13^C and δ^15^N for each cell. That is, each raster cell was given the same value for the turtle tissue-based δ^13^C and δ^15^N variance, but could have a unique variance from the kriging model. By summing these variances, we implicitly assume independence between the variance-generating process for observed δ^13^C and δ^15^N in tissues from a given foraging area and for the kriging model; we have no reason to believe that the foraging behavior of loggerheads would influence the way in which values are spatially interpolated in the kriging model. Because each raster cell had only one modeled value for δ^13^C and one for δ^15^N, and the modeling algorithm provided estimates only for the univariate standard errors (and not the bivariate covariances), we used the tissue-based estimates of covariance for the off-diagonals in the variance-covariance matrix.

We followed Wunder (2010) to create a spatially-explicit posterior probability distribution for each individual turtle in both the *calibration* (n = 227) and the *assignment* (n = 749) datasets. More specifically, we used the predicted values from the kriging model as the means and the variance-covariance matrix described above and estimated the posterior probability density function that each raster cell represented the foraging site origin from the measured tissue values for each turtle. All cells in each individual turtle raster were then normalized by the maximum value in that raster, creating a spatially-explicit distribution of cell values ranging from 0 to 1. This process resulted in an *assignment surface* for each individual turtle where a value of 1 represents the most probable foraging location and 0 the least probable^25^.

We summed the rescaled assignment surfaces for the 749 turtles in the *assignment* dataset (unknown foraging area) by year to identify the comparative strength of evidence for foraging regions across the study period. The resultant sums do not indicate the number of turtles that foraged at each given cell. The kriging models produced rasters with the same predicted values for δ^13^C and δ^15^N at more than one cell. Because we used the kriging predictions for each cell as the expected value for the cell in the assignment step, the assignment models also produced rasters where more than one cell was given the same assignment probability. Thus, when the rasters were rescaled to the maximum assignment value, more than one cell was given a value of 1. For this reason, the summed rasters should not be interpreted directly as the number of turtles foraging at each cell, but rather as the probable number of turtles that used the cell. These sums can be thought of as a probability-weighted index of the strength of evidence that a raster cell was the source of δ^13^C and δ^15^N values observed in the turtle tissues sampled each year (i.e., *an index of foraging importance*).

To identify the spatial origins of organic material for reproduction, we weighted the rescaled assignment rasters for an individual turtle using the known clutch size (number of eggs) and the estimated number of emergents from the nest for that turtle. That is, we computed the product of the number of eggs (or emergents) and the rescaled assignment probability value for every cell in the raster for each individual turtle. These rasters were then likewise summed by year to evaluate spatial patterns over time. These results can be informally considered as indices of the probable spatial distribution of contributions to reproduction (eggs or emergents) in the population (i.e., *indices of reproductive importance*). Hypothesis testing for no difference in mean numbers of nesting adults and emergence rate among identified hotspots for foraging and reproductive importance over the nine-year period was done using the Friedman rank sum test. Post-hoc hypothesis tests for pairwise similarity in mean numbers of nesting adults and emergence rate between hotspots were done using Conover tests adjusted with the Benjamini-Hochberg False Discovery Rate (FDR) method^65^.

All maps were newly created for this study using ArcGIS v. 10.2 (Esri Redlands, CA). We used R^66,67^ for all analyses, including estimating and applying all components of the assignment models, and the null hypothesis testing.

### Data Availability

The dataset analyzed in this study and the R scripts used to create the probability maps included in this published article are available as Supplementary Information files (Supplementary Data S3 and Supplementary Note S5).

## Electronic supplementary material

Supplementary information
