# Supplementary material for: Foraging and recruitment hotspot dynamics for the largest Atlantic loggerhead turtle rookery
Source: Sci Rep. 2017 Dec 4;7:16894. doi: 10.1038/s41598-017-17206-3 (PMC5715148; doi:10.1038/s41598-017-17206-3)
Supplement: Supplementary file 1 — Supplementary information [file 41598_2017_17206_MOESM1_ESM.pdf]

Supplementary materials for

Foraging and recruitment hotspot dynamics for the largest Atlantic loggerhead turtle rookery

Simona A. Ceriani [1, 2*], John F. Weishampel [2], Llewellyn M. Ehrhart [2], Katherine L. Mansfield [2], Michael B. Wunder [3]

1 Florida Fish and Wildlife Conservation Commission, Fish and Wildlife Research Institute, Saint Petersburg, Florida 33701, USA.

2 Department of Biology, University of Central Florida, Orlando, Florida 32816, USA

3 Department of Integrative Biology, University of Colorado Denver, Denver, Colorado 80204, USA

Corresponding author: * Simona A. Ceriani. Email: Simona.Ceriani@myfwc.com

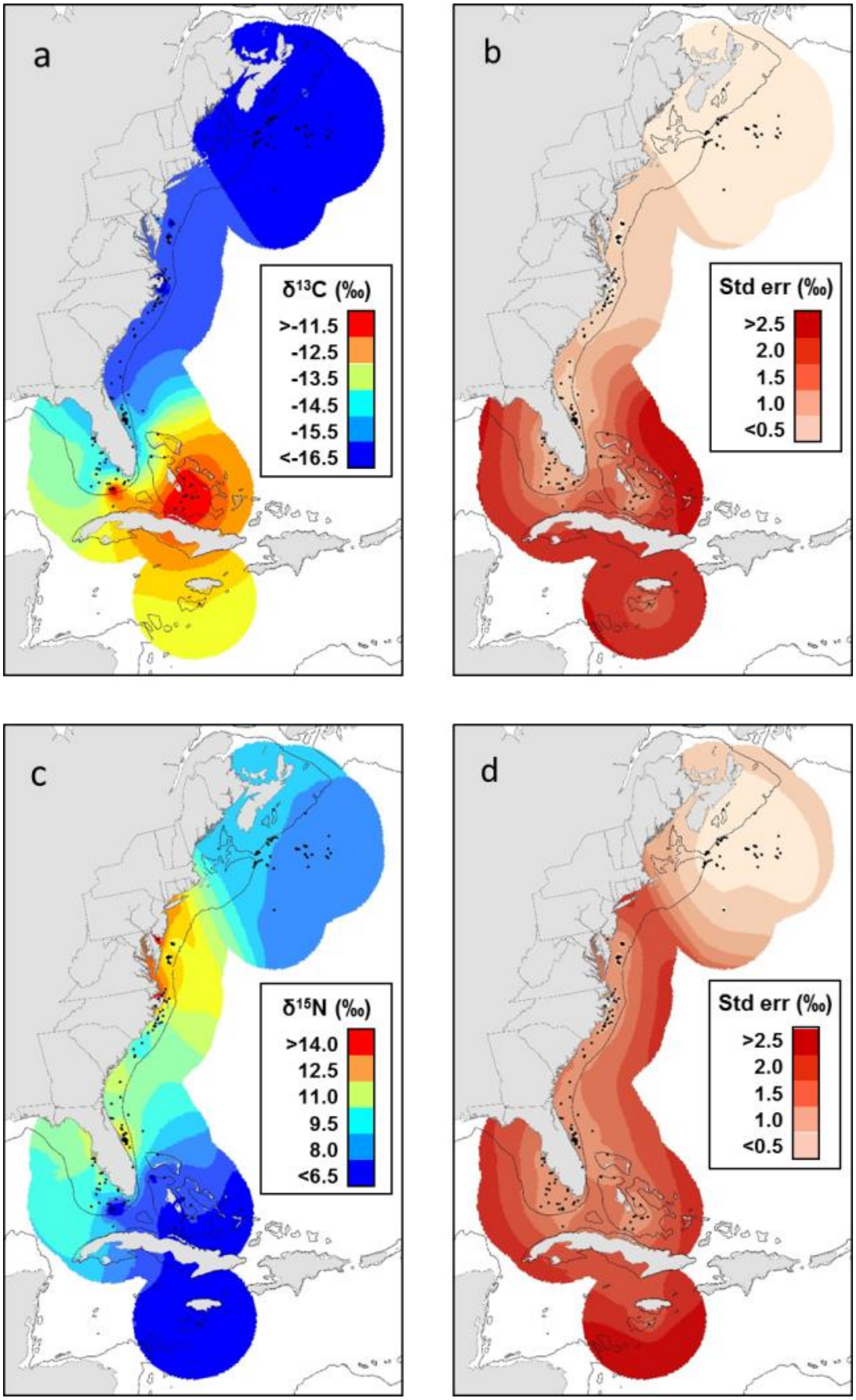

Supplementary Fig. S1. Isoscape models of $\delta^{13}C$ (a) and $\delta^{15}N$ (c) derived from loggerhead epidermal tissue (n=227) and associated standard error surfaces (b, d) based on cross validation

of observed and predicted values. Dots represent individual locations of foraging areas based on satellite telemetry and in-water captures for the 227 known-origin loggerheads sampled for SIA. Maps were created using ArcGIS v. 10.2 (http://www.esri.com/software/arcgis).

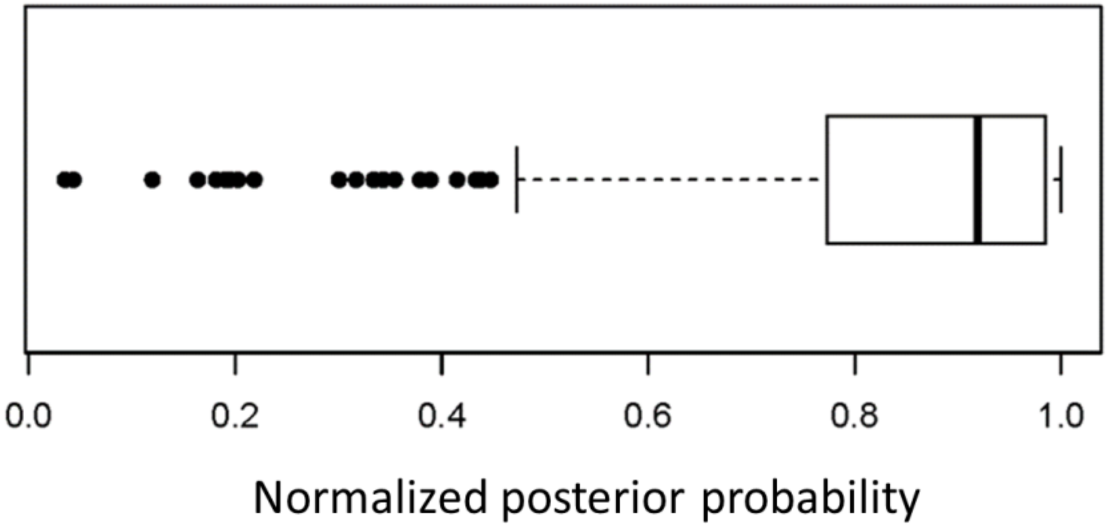

Supplementary Fig. S2. Validation results. Distribution of modeled normalized probability values for known-origins of turtles from satellite tagging and in-water captures.

Supplementary Data set S3. Assignment dataset (n=749 individual females): Turtle ID, year of sampling (Year), stable isotope values ($\delta^{13}C$, $\delta^{15}N$) measured directly from epidermis or derived from other tissues using published equations [1,2], clutch size (CS) and number of emergents (N_emergents).

TurtleID	Year	$\delta^{13}C$	$\delta^{15}N$	CS	N_emergents
1	2007	-16.28	11.64	106	73
2	2007	-8.05	7.74	122	74
3	2007	-9.96	7.01	83	65
4	2007	-13.72	8.86	97	49
5	2007	-15.93	12.04	92	70
6	2007	-16.78	15.83	104	57
7	2007	-10.97	8.94	110	81
8	2007	-12.59	7.41	87	0
9	2007	-16.20	9.63	89	21
10	2007	-9.54	7.88	127	92
11	2007	-11.77	8.58	156	139
12	2007	-8.76	4.88	106	82
13	2007	-14.67	10.38	107	92
14	2007	-17.12	13.08	140	112
15	2007	-16.51	8.64	136	26
16	2007	-15.47	13.22	121	102
17	2007	-10.12	7.46	140	90
18	2007	-14.95	12.25	97	66
19	2007	-14.09	13.78	122	74
20	2007	-14.11	11.32	65	50
21	2007	-10.39	8.77	106	102
22	2007	-10.93	6.92	116	105
23	2007	-9.60	7.83	138	129
24	2007	-13.38	11.28	87	45
25	2007	-15.76	12.77	148	19
26	2007	-16.08	12.02	120	60
27	2007	-11.10	7.37	108	63
28	2007	-9.21	8.44	124	112
29	2007	-15.41	12.49	99	89
30	2007	-8.65	6.47	124	0
31	2007	-12.76	12.49	127	0
32	2007	-16.03	14.28	135	107

33	2007	-10.95	9.95	118	112
34	2007	-11.29	9.34	111	98
35	2007	-12.72	8.16	91	87
36	2007	-14.19	7.35	123	96
37	2007	-13.27	7.92	130	84
38	2007	-13.19	12.69	90	33
39	2007	-17.70	12.72	121	111
40	2007	-11.27	8.23	108	69
41	2007	-10.25	6.64	160	109
42	2007	-12.54	8.20	68	54
43	2007	-17.10	12.39	87	45
44	2007	-15.15	15.25	127	0
45	2007	-7.98	6.52	150	67
46	2007	-9.31	6.98	168	16
47	2007	-17.95	13.74	146	123
48	2007	-12.37	11.96	122	61
49	2007	-16.02	12.45	117	35
50	2007	-10.90	9.33	113	44
51	2007	-14.92	11.74	121	111
52	2007	-9.46	8.28	126	48
53	2007	-10.25	6.77	155	139
54	2007	-15.80	12.04	147	139
55	2007	-15.35	14.52	118	27
56	2007	-15.56	10.51	111	31
57	2008	-16.13	13.04	87	15
58	2008	-15.94	12.08	85	0
59	2008	-9.73	8.52	126	97
60	2008	-11.40	7.23	144	51
61	2008	-15.64	15.27	123	0
62	2008	-16.65	14.77	102	27
63	2008	-16.01	16.73	125	0
64	2008	-14.88	10.16	92	70
65	2008	-10.87	8.30	121	64
66	2008	-12.11	7.39	119	23
67	2008	-11.20	6.94	160	129
68	2008	-15.66	12.13	104	96
69	2008	-10.31	8.64	119	100
70	2008	-15.21	16.33	119	108
71	2008	-16.61	13.08	130	105

72	2008	-14.45	12.91	86	0
73	2008	-14.22	11.88	120	81
74	2008	-14.13	12.05	118	96
75	2008	-12.34	8.34	112	98
76	2008	-14.62	12.63	100	89
77	2008	-10.32	8.54	110	90
78	2008	-15.97	12.79	122	95
79	2008	-17.11	14.91	113	14
80	2008	-16.05	12.49	141	98
81	2008	-10.25	6.05	151	117
82	2008	-14.01	11.60	123	0
83	2008	-16.07	16.70	108	102
84	2008	-15.65	17.26	79	21
85	2008	-15.70	12.01	137	78
86	2008	-16.01	15.33	99	0
87	2008	-14.18	9.14	109	0
88	2008	-16.09	17.05	142	99
89	2008	-14.84	16.70	116	81
90	2008	-13.93	11.13	93	78
91	2008	-15.47	16.49	130	39
92	2008	-12.18	8.12	87	58
93	2008	-13.55	8.58	115	52
94	2008	-10.88	6.80	145	126
95	2008	-11.18	6.78	126	0
96	2008	-16.26	14.75	166	49
97	2008	-15.40	11.81	109	90
98	2008	-9.13	7.30	136	48
99	2008	-13.89	11.74	93	25
100	2008	-12.05	7.30	100	75
101	2008	-15.00	17.90	134	119
102	2008	-15.84	11.82	126	92
103	2008	-14.77	12.09	95	89
104	2009	-15.01	11.27	91	73
105	2009	-16.92	11.84	117	99
106	2009	-16.7	11.85	154	0
107	2009	-10.15	7.11	87	16
108	2009	-10.48	7.15	128	112
109	2009	-15.45	10.57	116	90
110	2009	-9.72	7.46	124	95

111	2009	-15.43	13.79	134	110
112	2009	-14.08	11.07	102	67
113	2009	-16.76	12.05	103	60
114	2009	-15.53	12.07	97	0
115	2009	-15.37	10.66	75	55
116	2009	-14.18	11.03	105	73
117	2009	-15.61	12.15	71	18
118	2009	-15.73	13.82	133	27
119	2009	-14.60	11.84	98	8
120	2009	-14.80	11.70	101	0
121	2009	-16.91	9.08	81	0
122	2009	-9.57	8.31	132	0
123	2009	-9.87	6.80	159	71
124	2009	-11.64	8.55	111	97
125	2009	-16.79	16.96	125	7
126	2009	-14.37	10.66	124	52
127	2009	-17.51	10.17	94	0
128	2009	-11.63	7.25	117	51
129	2009	-9.98	7.80	148	46
130	2009	-15.56	15.12	99	37
131	2009	-14.95	11.32	75	43
132	2009	-15.83	11.99	93	6
133	2009	-8.66	8.27	154	52
134	2009	-16.01	12.91	87	82
135	2009	-12.91	11.74	108	0
136	2009	-14.37	12.35	92	0
137	2010	-17.06	16.01	137	66
138	2010	-17	16.2	118	84
139	2010	-16.41	17.02	129	79
140	2010	-19.10	9.77	95	52
141	2010	-10.44	7.46	154	53
142	2010	-11.41	6.79	151	124
143	2010	-10.31	6.91	151	72
144	2010	-15.96	16.68	103	46
145	2010	-15.93	16.70	101	42
146	2010	-16.20	10.37	120	88
147	2010	-12.94	8.79	93	49
148	2010	-8.25	6.02	159	72
149	2010	-14.34	10.65	114	64

150	2010	-15.78	10.77	107	85
151	2010	-10.48	7.35	144	6
152	2010	-15.39	14.72	133	66
153	2010	-15.84	11.81	114	79
154	2010	-14.57	10.05	107	90
155	2010	-15.16	11.11	101	76
156	2010	-14.30	11.63	31	25
157	2010	-8.72	7.23	80	0
158	2010	-9.36	8.16	80	69
159	2010	-13.97	11.00	109	48
160	2010	-16.72	10.35	86	38
161	2010	-11.65	7.64	132	0
162	2010	-14.90	10.90	89	43
163	2010	-14.98	11.92	81	54
164	2010	-12.85	7.83	108	67
165	2010	-17.07	13.31	162	62
166	2010	-11.75	6.43	142	124
167	2010	-9.72	5.20	95	60
168	2010	-9.84	7.83	150	72
169	2010	-8.67	6.15	140	47
170	2010	-11.45	10.39	111	109
171	2010	-9.79	11.86	140	122
172	2010	-8.37	8.47	141	89
173	2010	-10.00	6.75	127	93
174	2010	-16.30	16.50	145	131
175	2010	-10.04	6.48	116	81
176	2010	-10.50	6.06	85	0
177	2010	-14.05	12.57	140	83
178	2010	-14.22	12.70	93	44
179	2010	-14.35	12.76	88	65
180	2010	-14.41	12.34	115	72
181	2010	-14.62	12.34	66	63
182	2010	-15.03	12.74	75	67
183	2010	-16.10	12.37	91	59
184	2010	-17.56	12.56	145	28
185	2010	-15.42	12.27	107	32
186	2010	-16.24	12.49	122	0
187	2010	-14.38	12.14	76	67
188	2010	-14.58	12.25	77	65

189	2011	-16.85	12.48	127	72
190	2011	-15.10	13.24	147	48
191	2011	-10.93	6.82	160	129
192	2011	-9.40	6.34	155	87
193	2011	-9.58	7.22	105	52
194	2011	-9.93	8.69	94	0
195	2011	-13.10	10.98	131	9
196	2011	-14.80	5.67	150	63
197	2011	-13.31	11.01	155	135
198	2011	-12.01	8.89	118	0
199	2011	-15.37	9.88	101	89
200	2011	-15.76	13.77	155	77
201	2011	-10.74	8.77	74	61
202	2011	-9.49	9.20	128	79
203	2011	-9.08	8.08	161	135
204	2011	-17.36	13.74	128	99
205	2011	-9.24	6.36	124	110
206	2011	-17.12	10.26	123	114
207	2011	-15.17	11.56	99	21
208	2011	-13.53	7.34	122	36
209	2011	-10.13	7.87	108	89
210	2011	-15.87	10.17	71	67
211	2011	-16.04	6.25	172	139
212	2011	-16.40	11.87	109	0
213	2011	-15.42	12.13	145	55
214	2011	-14.90	10.56	96	92
215	2011	-15.27	11.34	92	32
216	2011	-15.96	11.97	96	51
217	2011	-10.88	11.31	122	48
218	2011	-15.45	13.80	119	36
219	2011	-12.16	9.56	103	27
220	2011	-15.82	9.52	125	116
221	2011	-15.52	16.86	106	0
222	2011	-9.76	6.62	127	106
223	2011	-10.85	8.74	106	104
224	2011	-9.92	8.76	127	114
225	2011	-16.20	11.63	93	74
226	2011	-11.64	7.69	117	65
227	2011	-15.36	14.13	125	90

228	2011	-16.03	15.40	148	63
229	2011	-10.32	6.25	138	100
230	2011	-10.43	6.26	158	0
231	2011	-12.96	11.79	76	0
232	2011	-14.65	12.76	110	0
233	2011	-13.32	11.73	174	110
234	2011	-14.93	12.54	112	15
235	2011	-10.36	12.01	132	52
236	2011	-14.81	12.05	158	136
237	2011	-14.39	12.13	87	0
238	2011	-14.43	12.23	78	0
239	2012	-13.60	11.36	117	113
240	2012	-15.38	10.79	100	87
241	2012	-15.21	16.88	84	65
242	2012	-15.06	9.94	91	73
243	2012	-13.55	7.49	117	102
244	2012	-11.09	6.32	109	78
245	2012	-12.29	7.36	132	74
246	2012	-9.41	6.20	123	106
247	2012	-9.25	8.69	118	47
248	2012	-11.15	6.43	55	32
249	2012	-16.93	12.17	73	63
250	2012	-15.61	9.98	126	106
251	2012	-11.50	6.10	116	115
252	2012	-15.61	11.40	90	70
253	2012	-16.48	10.27	103	36
254	2012	-14.89	13.26	82	59
255	2012	-14.69	14.13	68	66
256	2012	-16.22	13.08	62	57
257	2012	-11.29	9.34	123	117
258	2012	-11.69	7.98	142	130
259	2012	-14.69	12.18	86	73
260	2012	-15.85	12.33	84	50
261	2012	-15.88	12.06	131	0
262	2012	-16.75	13.01	90	78
263	2012	-12.05	8.92	128	105
264	2012	-16.91	9.29	111	93
265	2012	-16.77	15.32	110	50
266	2012	-16.44	12.07	73	72

267	2012	-16.85	11.15	115	91
268	2012	-17.80	12.34	90	59
269	2012	-15.44	12.63	80	78
270	2012	-16.13	12.55	112	71
271	2012	-15.25	12.04	104	78
272	2012	-15.66	12.41	96	15
273	2012	-10.09	6.60	125	114
274	2012	-11.57	7.95	111	84
275	2012	-15.06	8.22	82	27
276	2012	-9.79	6.72	123	106
277	2012	-14.68	8.00	119	113
278	2012	-9.47	5.79	81	80
279	2012	-16.36	14.50	102	94
280	2012	-16.21	13.59	80	72
281	2012	-16.42	12.63	79	60
282	2012	-15.10	11.89	88	74
283	2012	-16.57	10.45	102	94
284	2012	-17.79	12.50	51	19
285	2012	-17.69	9.09	97	0
286	2012	-16.21	9.85	98	72
287	2012	-15.58	17.49	159	66
288	2012	-15.06	12.55	94	0
289	2012	-16.73	12.11	78	14
290	2012	-15.15	12.62	73	57
291	2012	-17.26	15.97	129	53
292	2012	-15.06	12.93	79	52
293	2012	-16.40	13.34	116	31
294	2013	-12.51	12.19	122	66
295	2013	-15.92	10.08	71	46
296	2013	-11.31	8.53	135	127
297	2013	-13.97	13.46	85	44
298	2013	-14.94	11.67	129	102
299	2013	-8.32	8.60	136	129
300	2013	-8.00	6.29	138	117
301	2013	-10.89	5.87	148	142
302	2013	-8.01	7.90	54	29
303	2013	-7.99	6.54	141	96
304	2013	-17.96	10.80	63	30
305	2013	-12.82	9.13	61	0

306	2013	-16.09	16.71	105	99
307	2013	-10.29	7.01	102	99
308	2013	-17.77	19.49	138	80
309	2013	-15.88	11.08	121	87
310	2013	-15.93	12.99	134	123
311	2013	-15.37	12.88	142	141
312	2013	-17.63	13.74	138	59
313	2013	-8.40	6.50	106	80
314	2013	-16.01	12.71	94	0
315	2013	-12.11	8.55	114	109
316	2013	-17.04	9.15	149	112
317	2013	-9.50	4.27	127	96
318	2013	-13.81	10.57	105	102
319	2013	-13.75	8.49	89	85
320	2013	-15.36	12.43	79	40
321	2013	-15.27	14.74	99	4
322	2013	-14.81	13.92	140	124
323	2013	-14.53	9.94	119	114
324	2013	-15.46	9.90	90	76
325	2013	-16.59	10.54	45	18
326	2013	-15.99	12.64	94	0
327	2013	-15.28	10.26	105	100
328	2013	-16.36	15.36	100	77
329	2013	-8.68	6.25	39	6
330	2013	-16.46	12.19	124	0
331	2013	-16.45	13.69	60	0
332	2013	-16.86	15.03	120	98
333	2013	-14.47	10.90	82	74
334	2013	-12.47	6.32	180	108
335	2013	-14.90	12.39	78	66
336	2013	-13.92	8.96	121	74
337	2013	-14.66	12.74	107	99
338	2013	-17.40	14.13	117	61
339	2013	-16.96	17.98	72	40
340	2013	-17.37	11.89	110	103
341	2013	-15.22	11.75	98	94
342	2013	-19.92	10.33	108	65
343	2013	-14.53	9.82	110	103
344	2013	-7.02	3.69	98	94

345	2013	-16.39	10.27	113	63
346	2013	-12.02	9.08	104	99
347	2013	-14.41	10.00	99	93
348	2013	-7.91	5.52	115	111
349	2013	-8.56	4.04	124	109
350	2013	-10.85	3.58	94	88
351	2013	-15.98	9.17	26	16
352	2013	-15.05	9.46	96	91
353	2013	-16.90	13.01	22	3
354	2013	-10.64	7.11	84	78
355	2013	-11.57	8.86	28	0
356	2013	-11.24	4.58	129	78
357	2013	-14.94	11.88	91	59
358	2013	-15.54	12.10	80	62
359	2013	-8.38	6.82	91	59
360	2013	-9.29	6.70	92	56
361	2013	-16.44	15.06	111	97
362	2013	-12.99	9.70	90	44
363	2013	-10.43	6.63	87	59
364	2013	-15.38	12.29	159	149
365	2013	-16.52	14.38	127	65
366	2013	-11.53	6.79	149	137
367	2013	-10.95	6.32	125	48
368	2013	-15.85	11.87	135	63
369	2013	-11.33	8.28	95	65
370	2013	-9.93	4.56	89	63
371	2013	-16.76	9.33	118	96
372	2013	-16.67	12.39	93	49
373	2013	-16.49	15.37	130	118
374	2013	-17.44	9.76	118	88
375	2013	-17.35	10.64	125	114
376	2013	-9.39	6.00	111	56
377	2014	-11.59	8.51	96	91
378	2014	-16.22	12.02	135	112
379	2014	-13.09	8.66	77	47
380	2014	-15.23	12.79	100	90
381	2014	-8.70	6.47	104	69
382	2014	-10.55	9.83	139	126
383	2014	-16.63	12.84	107	0

384	2014	-14.35	11.39	82	70
385	2014	-12.55	11.18	94	88
386	2014	-15.45	12.06	112	95
387	2014	-14.80	12.59	105	92
388	2014	-10.33	7.54	89	75
389	2014	-9.13	7.23	102	94
390	2014	-8.64	5.85	130	95
391	2014	-14.45	11.84	102	86
392	2014	-8.89	4.74	106	76
393	2014	-10.51	4.82	85	78
394	2014	-8.25	6.53	106	103
395	2014	-14.86	11.58	136	125
396	2014	-13.98	12.99	109	105
397	2014	-14.85	12.94	92	89
398	2014	-14.10	13.29	76	61
399	2014	-15.99	14.01	85	84
400	2014	-14.43	11.15	106	82
401	2014	-14.97	12.22	128	110
402	2014	-11.86	5.12	86	84
403	2014	-11.31	7.65	122	120
404	2014	-8.33	5.47	151	122
405	2014	-10.56	8.25	136	120
406	2014	-12.30	10.08	99	74
407	2014	-13.80	11.41	102	0
408	2014	-8.98	8.84	82	0
409	2014	-12.85	9.55	117	101
410	2014	-9.81	5.42	67	54
411	2014	-12.21	8.32	98	92
412	2014	-10.28	6.33	59	58
413	2014	-5.72	2.96	63	25
414	2014	-10.80	8.50	130	119
415	2014	-15.49	12.77	97	64
416	2014	-9.41	7.90	112	35
417	2014	-14.75	12.78	148	65
418	2014	-21.60	7.88	73	26
419	2014	-10.47	7.30	110	102
420	2014	-14.81	13.07	94	79
421	2014	-16.14	12.78	98	85
422	2014	-10.44	8.28	116	113

423	2014	-12.29	5.41	65	62
424	2014	-10.24	8.31	113	80
425	2014	-15.83	6.89	113	87
426	2014	-14.44	12.94	93	79
427	2014	-9.75	6.62	105	97
428	2014	-13.42	13.01	115	66
429	2014	-16.52	15.82	106	87
430	2014	-8.62	6.07	122	59
431	2014	-11.37	7.77	126	0
432	2014	-8.37	6.39	140	94
433	2014	-15.07	12.77	94	79
434	2014	-10.55	4.10	71	47
435	2014	-9.35	6.01	97	76
436	2014	-11.10	8.30	119	87
437	2014	-15.22	11.57	116	66
438	2014	-15.72	12.56	83	11
439	2014	-14.51	13.59	88	79
440	2014	-11.36	4.10	73	15
441	2014	-15.60	11.62	150	29
442	2014	-13.82	9.04	106	78
443	2014	-15.19	12.30	92	80
444	2014	-12.60	8.68	128	100
445	2014	-14.45	10.66	78	49
446	2014	-16.22	10.54	96	42
447	2014	-16.39	13.95	100	22
448	2014	-8.86	9.08	86	7
449	2014	-10.12	4.69	89	82
450	2015	-15.31	16.11	113	90
451	2015	-10.40	6.97	103	49
452	2015	-17.26	15.69	132	75
453	2015	-10.44	6.44	118	104
454	2015	-9.84	8.72	121	109
455	2015	-15.93	11.68	98	26
456	2015	-9.61	6.45	71	56
457	2015	-10.81	6.26	133	108
458	2015	-17.49	10.77	83	80
459	2015	-16.35	12.02	78	64
460	2015	-10.76	6.05	129	119
461	2015	-12.57	6.40	148	40

462	2015	-14.91	12.04	140	112
463	2015	-16.55	12.30	141	59
464	2015	-12.71	6.72	119	113
465	2015	-11.10	6.90	111	0
466	2015	-9.65	8.56	106	68
467	2015	-15.25	14.02	91	70
468	2015	-16.64	10.16	102	89
469	2015	-15.94	10.34	98	85
470	2015	-12.11	7.13	128	96
471	2015	-15.60	12.41	74	45
472	2015	-14.69	12.98	68	53
473	2015	-13.61	8.75	156	147
474	2015	-16.15	11.78	139	138
475	2015	-11.77	7.69	95	89
476	2015	-16.00	14.61	67	65
477	2015	-15.96	11.11	81	69
478	2015	-10.26	8.45	104	58
479	2015	-15.59	12.80	91	81
480	2015	-11.32	5.91	121	95
481	2015	-16.56	15.22	111	101
482	2015	-16.12	12.42	114	54
483	2015	-15.00	10.95	79	67
484	2015	-17.00	12.47	109	109
485	2015	-11.16	7.21	92	86
486	2015	-12.67	8.25	127	121
487	2015	-10.14	7.58	94	61
488	2015	-14.63	11.61	117	41
489	2015	-18.37	17.34	87	86
490	2015	-14.03	7.90	94	91
491	2015	-15.46	11.74	94	75
492	2015	-16.15	15.14	118	99
493	2015	-9.84	6.38	139	128
494	2015	-14.87	12.63	72	30
495	2015	-14.68	11.76	82	73
496	2015	-18.97	9.40	99	77
497	2015	-10.11	7.55	102	84
498	2015	-8.87	6.77	123	119
499	2015	-14.23	10.09	63	53
500	2015	-13.20	8.66	135	115

501	2015	-11.09	6.02	105	101
502	2015	-16.51	11.35	76	73
503	2015	-10.74	6.11	129	113
504	2015	-16.73	10.37	80	53
505	2015	-14.95	11.66	116	111
506	2015	-10.71	7.97	95	92
507	2015	-13.07	7.99	140	109
508	2015	-17.53	8.70	105	101
509	2015	-15.81	13.29	90	87
510	2015	-15.60	11.87	102	90
511	2015	-17.13	9.70	117	81
512	2015	-16.50	15.20	79	75
513	2015	-15.54	11.15	52	32
514	2007	-12.34	8.67		
515	2007	-14.82	10.22		
516	2007	-17.28	12.34		
517	2007	-10.30	7.03		
518	2007	-10.73	6.90		
519	2007	-10.85	7.68		
520	2007	-18.09	13.49		
521	2008	-13.31	11.67		
522	2008	-10.59	7.42		
523	2008	-14.93	14.56		
524	2008	-14.98	9.86		
525	2008	-13.46	11.14		
526	2008	-13.98	10.90		
527	2008	-14.50	10.96		
528	2008	-12.86	7.97		
529	2008	-13.15	7.65		
530	2008	-14.31	10.85		
531	2008	-12.09	9.90		
532	2008	-14.01	10.30		
533	2008	-11.01	7.26		
534	2008	-14.10	11.14		
535	2008	-12.79	8.38		
536	2008	-14.90	11.58		
537	2008	-17.27	15.58		
538	2008	-11.95	8.28		
539	2008	-17.53	15.60		

540	2008	-10.42	6.37		
541	2008	-10.40	6.84		
542	2008	-15.80	13.56		
543	2008	-13.16	11.69		
544	2008	-13.03	10.22		
545	2009	-12.92	7.95		
546	2009	-14.90	13.89		
547	2009	-10.04	5.72		
548	2009	-13.37	6.80		
549	2009	-15.08	6.96		
550	2009	-15.59	11.29		
551	2009	-16.20	11.77		
552	2009	-15.05	10.84		
553	2009	-14.01	10.43		
554	2009	-10.98	8.28		
555	2009	-15.91	11.85		
556	2009	-11.14	8.27		
557	2009	-14.15	11.14		
558	2009	-8.46	8.12		
559	2009	-16.62	11.04		
560	2009	-14.42	11.81		
561	2009	-10.01	6.76		
562	2009	-17.75	9.66		
563	2009	-14.63	11.49		
564	2009	-10.51	7.83		
565	2009	-7.56	8.77		
566	2009	-9.23	8.62		
567	2009	-15.86	12.91		
568	2009	-13.86	12.00		
569	2009	-14.93	12.20		
570	2010	-14.44	12.98		
571	2010	-13.93	12.64		
572	2010	-14.08	13.06		
573	2010	-10.11	8.33		
574	2010	-14.88	11.84		
575	2010	-10.64	6.80		
576	2010	-10.20	6.93		
577	2010	-8.00	7.44		
578	2010	-9.38	8.10		

579	2010	-11.68	9.52		
580	2010	-9.96	8.70		
581	2010	-12.76	8.55		
582	2010	-15.93	15.53		
583	2010	-14.72	11.88		
584	2010	-16.20	12.24		
585	2010	-16.73	11.99		
586	2010	-17.77	9.96		
587	2010	-15.02	12.71		
588	2011	-16.08	15.33		
589	2011	-14.96	11.72		
590	2011	-16.95	13.18		
591	2011	-11.29	8.67		
592	2011	-15.98	13.39		
593	2011	-15.57	13.58		
594	2011	-10.12	8.08		
595	2011	-14.99	10.18		
596	2011	-15.59	12.07		
597	2011	-10.66	7.75		
598	2011	-16.26	11.77		
599	2011	-8.53	4.98		
600	2011	-10.09	7.66		
601	2011	-11.28	8.01		
602	2011	-9.12	7.79		
603	2011	-14.45	11.14		
604	2011	-7.88	7.62		
605	2011	-16.98	10.47		
606	2011	-17.56	14.63		
607	2011	-17.63	9.11		
608	2011	-11.93	8.18		
609	2011	-11.03	9.94		
610	2011	-16.01	12.99		
611	2012	-15.41	11.54		
612	2012	-17.38	15.68		
613	2012	-17.28	16.64		
614	2012	-16.40	16.15		
615	2012	-10.80	9.16		
616	2012	-16.14	11.98		
617	2012	-12.00	9.72		

618	2012	-14.89	9.44		
619	2012	-9.13	7.21		
620	2012	-15.85	14.11		
621	2012	-10.49	7.94		
622	2012	-11.11	9.29		
623	2012	-15.69	11.64		
624	2012	-9.32	8.71		
625	2012	-16.90	13.20		
626	2012	-13.50	8.58		
627	2012	-11.01	5.71		
628	2012	-11.11	5.73		
629	2012	-9.81	6.29		
630	2012	-10.16	6.29		
631	2012	-10.61	6.69		
632	2012	-11.84	6.96		
633	2012	-10.19	7.14		
634	2012	-11.60	7.23		
635	2012	-11.69	7.35		
636	2012	-9.85	7.36		
637	2012	-11.90	7.38		
638	2012	-10.25	7.42		
639	2012	-12.91	7.85		
640	2012	-16.74	8.16		
641	2012	-11.01	8.20		
642	2012	-13.43	8.46		
643	2012	-8.80	8.47		
644	2012	-9.75	8.58		
645	2012	-20.79	10.22		
646	2012	-18.92	10.35		
647	2012	-16.28	11.75		
648	2012	-16.21	11.99		
649	2012	-13.57	12.09		
650	2012	-16.83	12.54		
651	2012	-14.67	13.10		
652	2012	-14.25	13.13		
653	2012	-15.09	13.21		
654	2012	-14.40	13.36		
655	2012	-15.82	13.58		
656	2012	-18.22	13.82		

657	2012	-15.58	14.11		
658	2012	-15.58	14.76		
659	2013	-10.45	8.79		
660	2013	-16.26	11.96		
661	2013	-9.07	6.33		
662	2013	-15.17	13.24		
663	2013	-16.01	11.57		
664	2013	-9.53	8.66		
665	2013	-15.49	11.47		
666	2013	-10.96	8.57		
667	2013	-17.73	8.77		
668	2013	-8.98	7.40		
669	2013	-11.21	8.27		
670	2013	-14.76	11.00		
671	2013	-10.61	8.07		
672	2013	-8.79	3.66		
673	2013	-15.80	10.52		
674	2015	-15.47	11.57		
675	2015	-11.02	8.65		
676	2015	-10.26	7.38		
677	2015	-16.40	16.04		
678	2015	-14.46	9.37		
679	2015	-9.01	8.15		
680	2015	-16.45	14.66		
681	2015	-11.24	6.48		
682	2015	-15.61	15.76		
683	2015	-16.29	13.73		
684	2015	-17.27	10.63		
685	2015	-12.98	10.37		
686	2015	-10.23	7.92		
687	2015	-17.46	9.62		
688	2015	-16.26	14.37		
689	2015	-11.27	7.15		
690	2015	-11.42	6.77		
691	2015	-14.35	9.01		
692	2015	-14.42	12.66		
693	2015	-15.58	12.40		
694	2015	-16.73	11.85		
695	2015	-16.73	14.20		

696	2015	-10.39	5.46		
697	2015	-16.40	9.18		
698	2015	-10.89	8.08		
699	2015	-10.14	6.44		
700	2015	-16.68	12.77		
701	2015	-14.68	12.56		
702	2015	-16.42	13.74		
703	2015	-10.89	5.81		
704	2015	-16.21	16.01		
705	2015	-9.44	7.03		
706	2015	-11.38	6.01		
707	2015	-16.90	12.70		
708	2015	-15.83	9.38		
709	2015	-14.88	12.16		
710	2015	-17.07	11.27		
711	2015	-16.60	15.94		
712	2015	-14.43	12.84		
713	2015	-12.64	7.48		
714	2015	-14.43	9.37		
715	2015	-16.70	17.14		
716	2015	-14.63	10.38		
717	2015	-11.09	5.00		
718	2015	-15.83	14.11		
719	2015	-10.52	5.41		
720	2015	-16.97	12.88		
721	2015	-11.41	7.14		
722	2015	-10.60	6.43		
723	2015	-16.31	15.77		
724	2015	-16.89	8.66		
725	2015	-15.79	14.95		
726	2015	-16.14	13.91		
727	2015	-13.57	9.81		
728	2015	-17.11	14.81		
729	2015	-16.94	9.26		
730	2015	-14.61	13.34		
731	2015	-9.65	8.69		
732	2015	-10.49	8.56		
733	2015	-10.74	6.84		
734	2015	-9.33	7.34		

735	2015	-15.63	11.73		
736	2015	-11.05	7.09		
737	2015	-8.81	6.70		
738	2015	-8.92	7.91		
739	2015	-11.86	9.28		
740	2015	-16.47	10.19		
741	2015	-10.14	6.82		
742	2015	-11.16	6.43		
743	2015	-8.26	4.08		
744	2015	-16.71	16.76		
745	2015	-16.49	16.76		
746	2015	-15.59	13.86		
747	2015	-10.88	7.04		
748	2015	-13.87	11.12		
749	2015	-10.43	6.83		

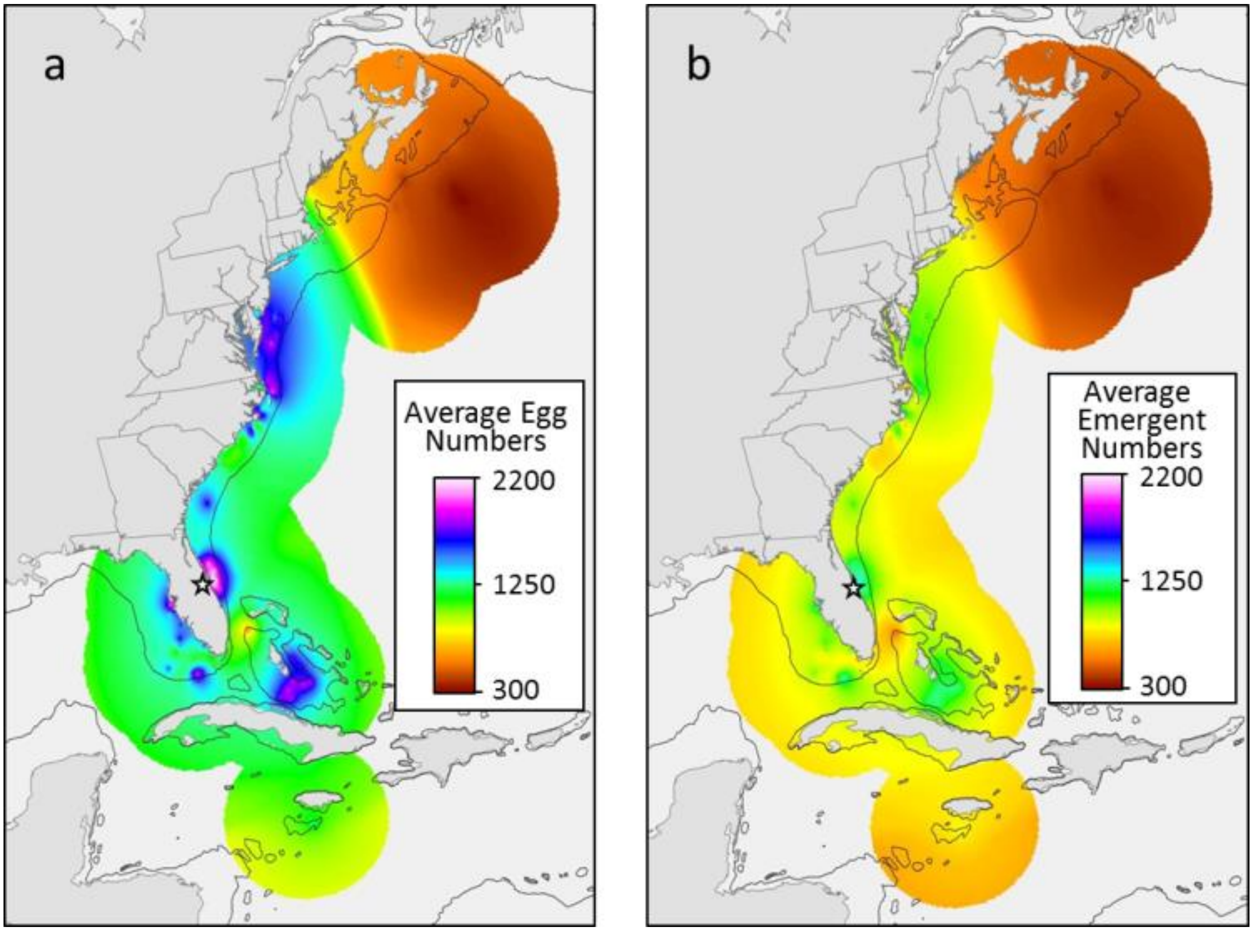

Supplementary Fig. S4. Population-level summary representing relative contribution of foraging regions to reproductive output at ACNWR based on estimated (a) egg and (b) emergent numbers averaged over nine years (2007-2015). Maps were created using ArcGIS v. 10.2 (http://www.esri.com/software/arcgis).

1 Ceriani, S. A. *et al.* Carry-over effects and foraging ground dynamics of a major loggerhead breeding aggregation. *Mar. Biol.* **162**, 1955-1968, doi:10.1007/s00227-015-2721-x (2015).

2 Ceriani, S. A., Roth, J. D., Ehrhart, L. M., Quintana-Ascencio, P. F. & Weishampel, J. F. Developing a common currency for stable isotope analyses of nesting marine turtles. *Mar. Biol.*, 257–2268 (2014).

Supplementary Note S5. R Scripts used to create the probability maps.

```
################################################################################
######
## turtle.assign.R
## July 2014, Denver, MBW
## AIMS: 1/ compute cell use probabilites for individual loggerhead turtles
##      2/ relate those to clutch size and emergent success for each individual
##      3/ summarize these results to infer origins of carbon for new turtles
################################################################################
######

# load libraries
library("rgdal")
library("raster")
library("maps")
library("maptools")
library("rasterVis")
library("mvnmle")
library("mixtools")

################################################################################
######
# Define new functions
################################################################################
######

# pdRaster: returns raster of probability density for each cell
# Assumes a bivariate normal probability distribution for 13C and 15N in each cell
# For computational efficiency, this function vectorizes isoscape raster
# x = observed isotope value for individual turtle (data)
# m = vector for mu; isoscape values for 13C and 15N
# v = vector for sigma^2; per-cell variance estimates
# r = correlation coefficient for 13C and 15N; stationary
# ras = raster extent; used to spatially constrain output
pdRaster <- function(x,m,v,r,ras) {
   pd <- 1/(2*pi*sqrt(v[,1])*sqrt(v[,2])*sqrt(1-r^2))*exp(-(1/(2*(1-r^2)))*
      ((x[1]-m[,1])^2/v[,1]+(x[2]-m[,2])^2/v[,2]-(2*r*(x[1]-m[,1])*
      (x[2]-m[,2]))/(sqrt(v[,1])*sqrt(v[,2]))))
   pdras <- setValues(ras,pd)
   return(pdras)
}

# qtlRaster: returns raster identifying upper quantiles across raster values
qtlRaster <- function(ras,q=0.95) {
  qras <- ras >= quantile(ras,probs=q)
  return(qras)
}
```

```
# llraster: returns raster of log likelihoods for each cell
llRaster <- function(x,m,v,r,ras) {
  n <- nrow(x)
  k <- ncol(x)
  for (i in 1:nrow(m)) {
    mu <- m[i,]
    cv <- matrix(v[i,1],r,v[i,2],r,nrow=2)
    for (s in 1:n) {
      xs <- x[s,]
      kernSum  <- crossprod(x,solve(cv))%*%x
    }
    ll[i] <- - (n * k / 2) * (log(2*pi)) - (n/2) * log(abs(cv)) - kernSum/2
  }
  llras <- setValues(ras,ll)
  return(llras)
}

################################################################################
######
## Data input: read in and process data
################################################################################
######

# Turtle Data
########################################################################
# Load data for turtles with known-origin tissue
turtles.known<-read.csv("./data/knownturtles.csv")

# estimate variance-covariance at known foraging areas
can.vcm<-mlest(cbind(turtles.known[turtles.known$ForArea=="CAN",]$d13c,
                     turtles.known[turtles.known$ForArea=="CAN",]$d15n))$sigmahat
mab.vcm<-mlest(cbind(turtles.known[turtles.known$ForArea=="MAB",]$d13c,
                     turtles.known[turtles.known$ForArea=="MAB",]$d15n))$sigmahat
sab.vcm<-mlest(cbind(turtles.known[turtles.known$ForArea=="SAB",]$d13c,
                     turtles.known[turtles.known$ForArea=="SAB",]$d15n))$sigmahat
snwa.vcm<-mlest(cbind(turtles.known[turtles.known$ForArea=="SNWA",]$d13c,
                      turtles.known[turtles.known$ForArea=="SNWA",]$d15n))$sigmahat
keys.vcm<-mlest(cbind(turtles.known[turtles.known$ForArea=="Keys",]$d13c,
                      turtles.known[turtles.known$ForArea=="Keys",]$d15n))$sigmahat
swfl.vcm<-mlest(cbind(turtles.known[turtles.known$ForArea=="SWFL",]$d13c,
                      turtles.known[turtles.known$ForArea=="SWFL",]$d15n))$sigmahat

# compute average among-turtle correlations and variances for foraging areas
rho <- mean(c(can.vcm[2,1]/(sqrt(can.vcm[1,1])*sqrt(can.vcm[2,2])),
            mab.vcm[2,1]/(sqrt(mab.vcm[1,1])*sqrt(mab.vcm[2,2])),
```

```
            sab.vcm[2,1]/(sqrt(sab.vcm[1,1])*sqrt(sab.vcm[2,2])),
            snwa.vcm[2,1]/(sqrt(snwa.vcm[1,1])*sqrt(snwa.vcm[2,2])),
            keys.vcm[2,1]/(sqrt(keys.vcm[1,1])*sqrt(keys.vcm[2,2])),
            swfl.vcm[2,1]/(sqrt(swfl.vcm[1,1])*sqrt(swfl.vcm[2,2]))))
var.c <- mean(c(can.vcm[1,1],mab.vcm[1,1],sab.vcm[1,1],snwa.vcm[1,1],
             keys.vcm[1,1],swfl.vcm[1,1]))
var.n <- mean(c(can.vcm[2,2],mab.vcm[2,2],sab.vcm[2,2],snwa.vcm[2,2],
             keys.vcm[2,2],swfl.vcm[2,2]))
vcvm <- matrix(c(var.c,rho,rho,var.n),nrow=2,byrow=T)

# load data for turtles with unknown foraging areas (to be assigned)
turtles <- read.csv("./data/turtles.csv")
turtles$year <- as.factor(turtles$year)
turtles.kfate <- turtles[!is.na(turtles$clutch),] # subset with known nest fates

# GIS
data  ##########################################################################
# carbon and nitrogen models
d13c<-raster("./data/d13c.txt")
d15n<-raster("./data/d15n.txt")

# associated kriging error estimates
d13c.se<-raster("./data/d13c_se.txt")
d15n.se<-raster("./data/d15n_se.txt")

# reproject all to lat lon
data(wrld_simpl)
crs(d13c)<-projection(wrld_simpl)
crs(d13c.se)<-projection(wrld_simpl)
crs(d15n)<-projection(wrld_simpl)
crs(d15n.se)<-projection(wrld_simpl)

# make a mask
rc<-setValues(d13c,NA) # make the dummy raster
rmask<-rasterize(wrld_simpl,rc) # make the mask
rmask<-is.na(rmask)
rmask<-reclassify(rmask,cbind(0,NA))

# mask all rasters
rc<-mask(x=d13c,mask=rmask)
rc<-trim(rc)
rcse<-mask(x=d13c.se,mask=rmask)
rcse<-trim(rcse)
rn<-mask(x=d15n,mask=rmask)
rn<-trim(rn)
rnse<-mask(x=d15n.se,mask=rmask)
```

```
rnse<-trim(rnse)

# get values from isoscapes for mean of model
mu<-cbind(getValues(rc),getValues(rn))

# combine turtle sampling and kriging model variances
vars<-cbind(getValues(rcse)^2+var.c,getValues(rnse)^2+var.n)

# clean up
rm(d13c,d13c.se,d15n,d15n.se,var.c,var.n,can.vcm,mab.vcm,
   sab.vcm,snwa.vcm,keys.vcm,swfl.vcm,rcse,rnse,rn)

################################################################################
#####
## Fit models
################################################################################
#####

# calibration (known origin) turtle probs
norm.prob.cal <- stack()
origin.val <- NULL
for (i in seq(along=turtles.known[,2])) {
  tm <- c(turtles.known$d13c[i],turtles.known$d15n[i])
  xy <- data.frame(x=turtles.known$lon[i],y=turtles.known$lat[i])
  rasta <- pdRaster(tm,mu,vars,rho,rc)
  np <- rasta/cellStats(rasta,max)
  origin.val[i] <- extract(np,xy)
  norm.prob.cal <- stack(norm.prob.cal, np)
}

# unknown origin turtle probs
norm.prob <- stack()
for (i in seq(along=turtles[,1])) {
    tm <- c(turtles$carbon[i],turtles$nitrogen[i])
    rasta <- pdRaster(tm,mu,vars,rho,rc)
    np <- rasta/cellStats(rasta,max)
    norm.prob <- stack(norm.prob, np)
}

# cleanup
rm(i,tm,rasta,np,vcvm,rc,rho,rmask,wrld_simpl,mu,vars,xy)

# annual summaries for known nesting fate subset
forage <- stack()
ann.forage <- stack()
ann.clutch <- stack()
```

```
ann.emerge <- stack()
ann.prod <- stack()
for (i in seq(levels(turtles.kfate$year))) {
  idx <- which(turtles.kfate$year==levels(turtles.kfate$year)[i])
  f <- sum(subset(norm.prob,idx))
  #fn <- f/length(idx)
  print(f)
  c <- sum(subset(norm.prob,idx)*turtles$clutch[idx])
  e <- sum(subset(norm.prob,idx)*turtles$emerge[idx])
  p <- 1-(c-e)/c
  forage <- stack(forage, f)
  ann.forage <- stack(ann.forage, f)
  ann.clutch <- stack(ann.clutch, c)
  ann.emerge <- stack(ann.emerge, e)
  ann.prod <- stack(ann.prod, p)
  writeRaster(f,paste("./results/rasters/",levels(turtles$year)[i],
                      "forage.tif",sep=""),format="GTiff",overwrite=T)
  writeRaster(c,paste("./results/rasters/",levels(turtles$year)[i],
                      "clutch.tif",sep=""),format="GTiff",overwrite=T)
  writeRaster(e,paste("./results/rasters/",levels(turtles$year)[i],
                      "emerge.tif",sep=""),format="GTiff",overwrite=T)
  writeRaster(p,paste("./results/rasters/",levels(turtles$year)[i],
                      "produc.tif",sep=""),format="GTiff",overwrite=T)
}
names(ann.forage) <- paste("year",levels(turtles.kfate$year),sep=" ")
names(ann.clutch) <- paste("year",levels(turtles.kfate$year),sep=" ")
names(ann.emerge) <- paste("year",levels(turtles.kfate$year),sep=" ")
names(ann.prod) <- paste("year",levels(turtles.kfate$year),sep=" ")
rm(i,idx,f,fn,c,e,p)

################################################################################
#####
## summary plots
################################################################################
#####

## Relative importance of foraging areas
pdf("./results/forage.pdf")
f <- stack(sum(norm.prob)/749, sum(forage)/513)
f <- trim(f)
names(f) <- c('all','known fate')
spplot(f,col.regions=terrain.colors(20),
       main="Proportional importance of forage areas")
dev.off()
rm(f)
```

```r
## Eggs and emergents on same plot, same scale
pdf("./results/productivity.pdf")
s <- stack(sum(ann.clutch), sum(ann.emerge))
s <- trim(s)
names(s) <- c('eggs','emergents')
spplot(s,col.regions=terrain.colors(20),
       main="All years combined")
dev.off()
rm(s)

## Standardized difference between eggs and emergents
pdf("./results/netproductivity.pdf")
spplot(mean(ann.prod),col.regions=rev(terrain.colors(20)),
       main='Mean net proportional productivity:
       1-(eggs-emerge)/(eggs)')
dev.off()

## annual foraging on same plot, same scale
pdf("./results/foraging.ann.pdf")
spplot(ann.forage,col.regions=terrain.colors(20),
       main="Probable Number of Turtles Foraging in a Cell")
dev.off()

## annual productivity on same plot, same scale
pdf("./results/productivity.ann.pdf")
spplot(ann.prod,col.regions=rev(terrain.colors(20)),
       main="Annual net proportional productivity:
       1-(eggs-emerge)/eggs")
dev.off()
```
